# Chemotherapeutic and targeted drugs-induced immunogenic cell death in cancer models and antitumor therapy: An update review

**DOI:** 10.3389/fphar.2023.1152934

**Published:** 2023-04-21

**Authors:** Jiaqi Zhai, Xi Gu, Yang Liu, Yueting Hu, Yi Jiang, Zhenyong Zhang

**Affiliations:** Department of Oncology, Shengjing Hospital of China Medical University, Shenyang, Liaoning, China

**Keywords:** immunogenic cell death (ICD), damage-related molecular patterns (DAMPs), cancer chemoimmunotherapy, antitumor immunity, chemotherapeutic drug

## Abstract

As traditional strategies for cancer treatment, some chemotherapy agents, such as doxorubicin, oxaliplatin, cyclophosphamide, bortezomib, and paclitaxel exert their anti-tumor effects by inducing immunogenic cell death (ICD) of tumor cells. ICD induces anti-tumor immunity through release of, or exposure to, damage-related molecular patterns (DAMPs), including high mobility group box 1 (HMGB1), calreticulin, adenosine triphosphate, and heat shock proteins. This leads to activation of tumor-specific immune responses, which can act in combination with the direct killing functions of chemotherapy drugs on cancer cells to further improve their curative effects. In this review, we highlight the molecular mechanisms underlying ICD, including those of several chemotherapeutic drugs in inducing DAMPs exposed during ICD to activate the immune system, as well as discussing the prospects for application and potential role of ICD in cancer immunotherapy, with the aim of providing valuable inspiration for future development of chemoimmunotherapy.

## 1 Introduction

In past decades, chemotherapy drugs, have played significant roles in inhibiting tumor growth as the main strategy for treatment of malignant tumors. Further, treatment approaches combining chemotherapy and immunotherapy are among the most effective cooperative strategies and have made considerable contributions to tackling drug resistant tumor cells (Vanneman and Dranoff, 2012; Smyth et al., 2016). Complex immune components in the tumor microenvironment influence immunomodulatory effects on tumors, and may interfere with the therapeutic effects of chemotherapy drugs (Salmon et al., 2019), and additional chemoimmunotherapy regimens with potential anti-cancer effects have been discovered based on this mechanism (Galluzzi et al., 2019; Roumenina et al., 2019). Chemoimmunotherapy is considered a cutting-edge anti-tumor strategy (Galluzzi et al., 2015), and several preclinical and clinical studies have demonstrated that some chemotherapy agents can induce ICD (Vanmeerbeek et al., 2020). ICD is a form of regulated cell death typically driven by stress (Galluzzi et al., 2017), including cell stress and cell death accompanied by exposure, active secretion, or passive release of large numbers of DAMPs, such as calreticulin (CRT), adenosine triphosphate (ATP), heat shock protein (HSP), and high mobility group box 1 (HMGB1) (Garg et al., 2010; Krysko et al., 2012; Boada-Romero et al., 2020). Apoptotic cells release their contents, including DAMPs, which act as hazard signals that produce immunostimulatory effects, including recruitment and activation of various immune cells, such as neutrophils and macrophages (Nagata and Tanaka, 2017). ICD can both effectively activate immune responses and trigger tumor-specific adaptive immunity, which is crucial for stimulating dysfunctional anti-tumor immunity (Inoue and Tani, 2014). DAMPs can interact with the immune system, thereby altering immunogenic outcomes, as well as regulating the types of cell death that occur (Yatim et al., 2017). Unlike the swelling and rupture observed during necrosis, apoptotic cells are rapidly engulfed by macrophages under normal circumstances, which is also considered an immunogenic event (Obeid et al., 2007). Increasing evidence supports that the adaptive immune mechanisms initiated by malignant cells undergoing ICD are related to the release and detection of DAMPs, which interact with homologous pattern recognition receptors on innate immune cells, leading to immune cell activation and maturation, and consequent effective anti-cancer adaptive immune responses (Garg et al., 2014; Galluzzi et al., 2017). DAMPs have indispensable roles in cancer treatment by interacting with the immune system, as demonstrated by novel studies into DAMPs exposure/secretion, which have helped to identify new drugs that can induce ICD (Garg et al., 2010). ICD-related cellular stressors exploited for clinic treatment include, but are not limited to: 1) therapeutic oncolytic viruses (Brown et al., 2017; Fend et al., 2017); 2) conventional chemotherapy drugs, such as anthracyclines [doxorubicin (DOX), mitoxantrone (MTX) etc.] (Obeid et al., 2007; Fucikova et al., 2011), and DNA damaging agents [cyclophosphamide (CPA), platinum derivatives, but excluding cisplatin] (Schiavoni et al., 2011; Kopecka et al., 2018; Limagne et al., 2019; Wang et al., 2019; Yamazaki et al., 2020), proteasome inhibitors [bortezomib (BTZ)] (Spisek et al., 2007; Gulla et al., 2021), and paclitaxel (PTX) (Lau et al., 2020); 3) targeted anti-cancer drugs (cetuximab, crizotinib, ceritinib, and ibrutinib) (Sagiv-Barfi et al., 2015; Pozzi et al., 2016; Goel et al., 2017; Liu et al., 2019; Petroni et al., 2020; Petrazzuolo et al., 2021); and 4) various physical therapies (radiotherapy, external phototherapy, and photodynamic therapy, etc.) (Gomes-da-Silva et al., 2018; Tatsuno et al., 2019; Choi et al., 2021; Vaes et al., 2021).

Over the decades, two standards have been established to identify genuine ICD inducers *in vivo* (Garg et al., 2017a). First, ICD inducers must show superior therapeutic effect when used to treat tumors in mice with normal immune function compared with those with low immune function (Vesely et al., 2011; Garg et al., 2014; Kepp et al., 2014). Second, *in vitro*, cancer cells succumbing to genuine ICD inducers can vaccinate syngeneic immunocompetent hosts and fight subsequent attack with living cancer cells of the same type (Garg et al., 2016); however, it is not possible to discriminate genuine ICD inducers and chemotherapeutic drugs with immunostimulatory effects (Galluzzi et al., 2016a; Galluzzi et al., 2016b). Hence, the gold-standard for identifying instances of ICD relies more on the second approach in vaccination settings (Kepp et al., 2014). In recent years, chemoimmunotherapy has attracted increasing attention because of its promising prospects; many successful antitumor treatments can benefit from effective induction of tumor cell ICD. Further, numerous studies have confirmed that some chemotherapeutic agents can induce ICD to enhance tumor cell immunogenicity (Inoue and Tani, 2014; Vanmeerbeek et al., 2020). In this review, we focus on the main applications of ICD induced by chemotherapy and targeted drugs, and provide an update on progress in anti-tumor therapy with several specific drug types. The aim of this review is to provide valuable insights applicable to cancer immunotherapy.

## 2 ICD induced by chemotherapy

Gold-standard approaches to prediction of the ICD-inducing capacity of chemotherapeutic agents appear to rely on CRT exposure, ATP secretion, and HMGB1 release by human cancer cells (Galluzzi et al., 2015). CRT is a Ca^2+^ binding protein which mainly localizes to the endoplasmic reticulum (ER), and has various biological functions, including regulating calcium signal, participating in glycoprotein synthesis, and regulating gene expression (Shaffer et al., 2005). CRT is a recognized “eat me” signal on the surface of tumor cells. It can form a bridging complex with CD91 molecule on the surface of phagocytes to initiate clearance (Vandivier et al., 2002) Besides, when cells are in a pre-apoptotic stage, CRT is translocated to the cell periphery by immunogenic dead cells, along with ERp57. Once the CRT/ERp57 molecular complex is co-transferred and exposed on the cell surface, it promotes phagocytosis by dendritic cells (DCs) (Krysko et al., 2012; Galluzzi et al., 2020; Fucikova et al., 2021). In addition, CD47 on tumor cell membrane can inhibit the phagocytosis of DC to tumor cells (Chao et al., 2010). As the signal of “do not eat me”, when CD47 is blocked, the phagocytosis of macrophages is induced (Zhang et al., 2016). In the early ICD, CRT was exposed to the cell surface, accompanied by a significant decrease in CD47 expression. The coordination between the two signals can further trigger immunogenicity. These signals must be considered in order to achieve the better anti-tumor response (Feng et al., 2019). There is evidence that the molecular activity of ICD induced by anticancer drugs may be related to activation of an ER stress-mediated CRT expression pathway, thus inducing immunogenic apoptosis of cancer cells (Xu et al., 2017).

Another sign of ICD is secretion of ATP by dead cancer cells, which is considered to be a ‘find me’ signal (Martins et al., 2009). In most cases, secretion of large amounts of ATP by stressed cells is key to extracellular ATP-mediated immunostimulation and related to the functional autophagy response (Michaud et al., 2011). Extracellular ATP from cancer cells that undergo ICD mediates immune system chemotaxis by binding to purinergic receptor P2Y2 (P2RY2), and promoting the secretion of interleukin 1 β (IL-1β) and interleukin-18 (IL-18) by activating inflammatory corpuscles (Kepp et al., 2021). After the inflammasome is activated, newly-recruited DC precursors undergo maturation in response to ATP, and their ability to recognize and present tumor antigen is enhanced, thereby initiating adaptive anti-cancer immunity (Michaud et al., 2011).

In the late stages of apoptosis, when cells are damaged and disrupted, HMGB1 released from nuclei can be detected by enzyme-linked immunosorbent assay (Bell et al., 2006). HMGB1 is a key nuclear component of non-histone chromatin binding that is passively released by dead cells (Gardella et al., 2002). HMGB1 can combine with Toll-like receptor 4 (TLR4) on the cell membrane of DC, and transmit signals to stimulate DC maturation, thus mediating immune stimulation (Apetoh et al., 2007). When DAMPs combine with specific receptors, they can promote and recruit antigen presenting cells, resulting in cross-presentation of apoptosis-associated antigens to CD8^+^ cytotoxic T lymphocytes (CTLs), which secrete IL-1β. γδ T lymphocytes, which produce interleukin-17 (IL-17) are also associated with the subsequent adaptive immune response (Mattarollo et al., 2011; Kepp et al., 2014). As major mediators of tumor cell killing, CTLs have vital roles in cancer treatment (Farhood et al., 2019). Activated CTLs mainly kill target cells through granule exocytosis and Fas ligand (FasL)-mediated apoptosis induction, thus achieving tumor clearance (Russell and Ley, 2002).

**FIGURE 1:**
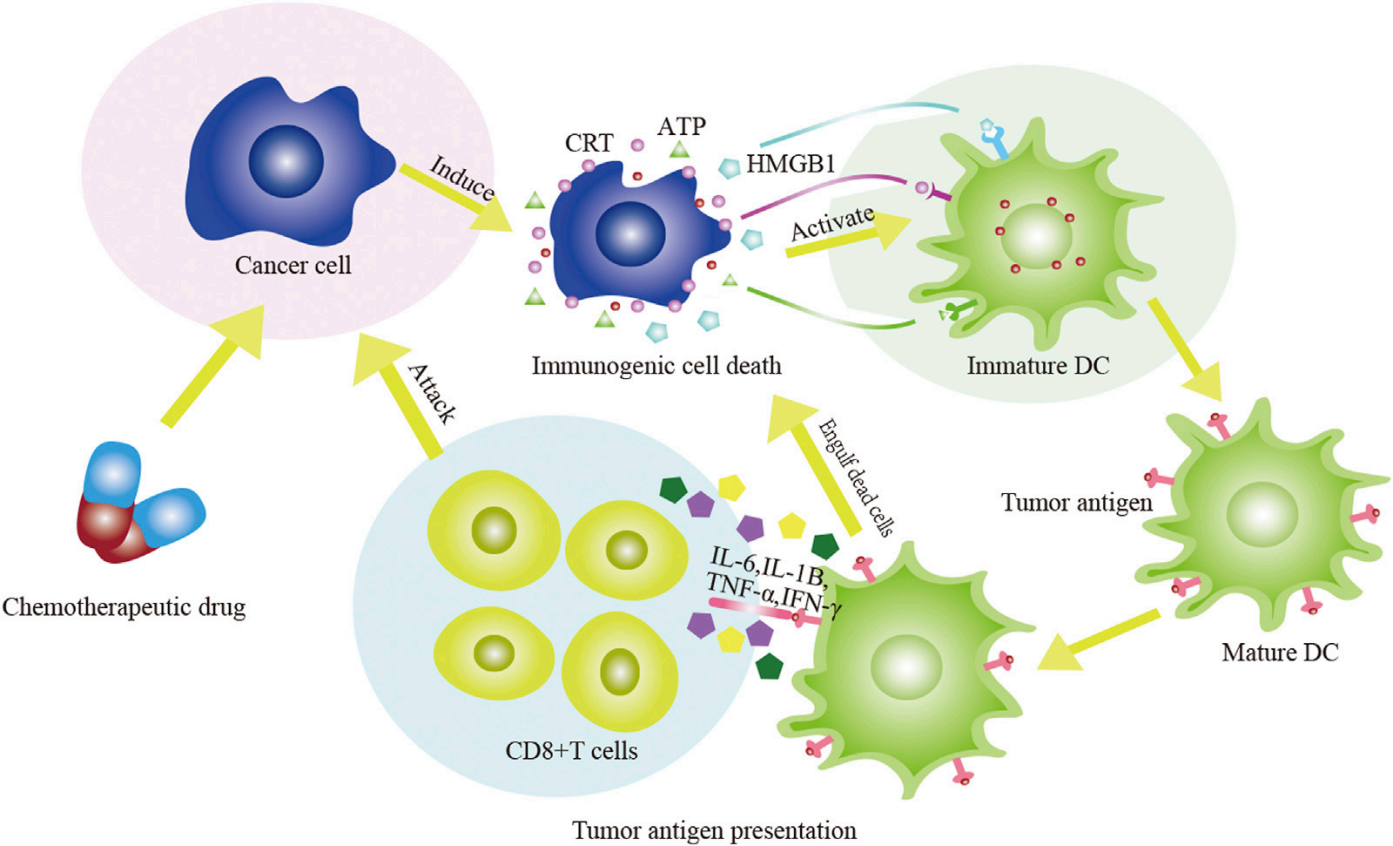
The diagram of chemotherapeutic drugs act on tumor cells to induce ICD. The chemotherapeutic drugs stimulate DC to phagocytose antigens and induce DC to mature through the release of a large number of damps. The effect of DC on T cells includes two aspects: DC directly acts on CTL; DC acts on CTL again by secreting IL-6, IL-1β, IFN-γ and TNF-α. The adaptive immune response is subsequently activated and the anti-tumor effect of chemotherapy drugs are enhanced.

Hence, ICD-inducing chemotherapy drugs can exert their effects both through their chemotherapeutic activity and by synergistic tumor cell killing through ICD-activated anti-tumor immune responses, thereby achieving better therapeutic effects (Figure 1; Table1). In addition, interaction between actively or passively released annexin A1 (ANXA1) and formyl peptide receptor 1 (FPR1) also contributes to anti-cancer immune responses to chemotherapy (Vacchelli et al., 2015). Further, immunostimulatory cytokines, such as type I interferon (IFN), have important roles in cancer treatment (Vanpouille-Box et al., 2017; Cauwels et al., 2018; Sprooten et al., 2019). Active secretion of chemokines, such as chemokine (C-X-C motif) ligand 1 protein (CXCL1), C-C motif ligand 2 (CCL2), and chemokine (C-X-C motif) ligand 10 protein (CXCL10), as well as passive release of nucleic acids, are also ICD-related factors driven by chemotherapy (Garg et al., 2017b).

**TABLE 1:** The chemoreagents involved in the ICD induction.

ICD inducers	Cancer type	Target	Effect	Ref
DOX	Breast cancer	Oncolytic peptide LTX-315	Enhance the infiltration of cytotoxic CD8 T cells	Camilio et al. (2019)
DOX	HCC	Icaritin	Exacerbates mitophagy and apoptosis and improve the anti-tumor effect of ICD	Yu et al. (2020)
DOX	Breast cancer	PEG-FA-Lip	Effectively arouse T cell immune response and inhibit lung metastasis	Deng et al. (2019)
Pegylated liposomal DOX	Ovariancancer	Motolimod	Promote the activation of immune cell biomarkers and increase t cell infiltration	Monk et al. (2017)
OXA	Colorectal cancer	Bacterial ghosts	Enhance the induction of ICD	Groza et al. (2018)
Amphiphilic OXA prodrug constructed liposomes	Colorectal cancer	Metformin	Help to trigger ICD together to enhance the anti-tumor effect	Song et al. (2022)
OXA prodrug	Colorectal cancer, breast cancer	PEGylated photosensitizer	Triggered a powerful anti-tumor immune response	Zhou et al. (2019)
OXA	NSCLC and fibrosarcoma	Thiostrepton	Enhance the induction of ICD	Qinyang Wang et al. (2020)
BTZ	Multiple myeloma	STING Agonists	Promote anti–multiple myeloma immune response	Gulla et al. (2021)
BTZ	Colon and colorectal cancer	Ionizing Radiation	Enhance T cell activity and anti-tumor immune attack	Cacan et al. (2015)
BTZ	Multiple myeloma	Geranylgeranyl diphosphate synthase inhibitor	enhance activation of ICD markers	Haney et al. (2022)

### 2.1 Anthracyclines

**FIGURE 2:**
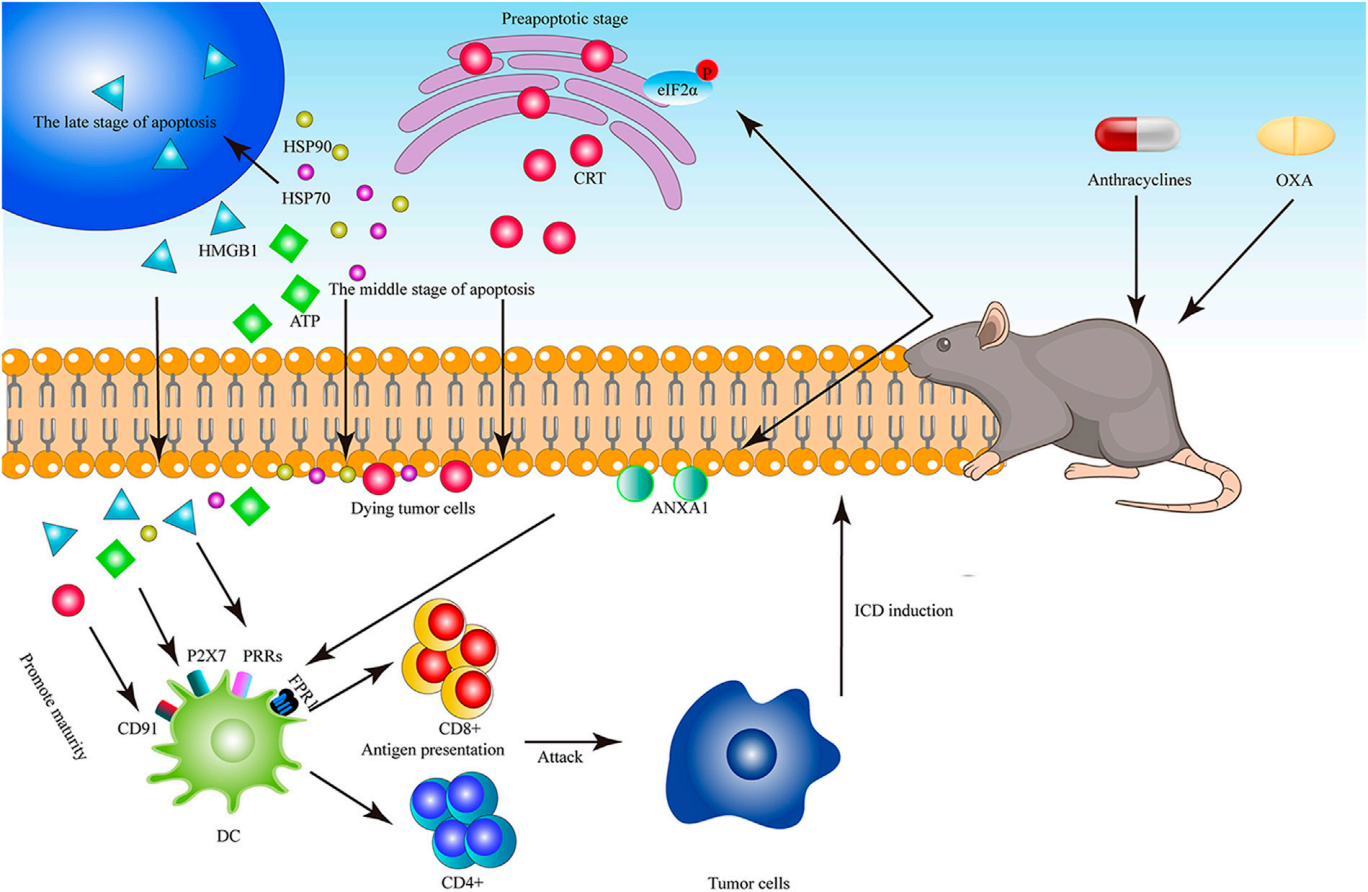
The diagram of Anthracyclines and OXA to induce ICD. Anthracyclines and OXA cause phosphorylation of EIF2A in dying tumor cells, and induce CRT translocation in the early stage of apoptosis, ATP secretion, HSP70 release in the middle stage of apoptosis and HMGB1 release in the late stage of apoptosis. CRT is combined with CD91, ATP is combined with P2x7, and HMGB1 is combined with TLR4. In addition, ANXA1 on the surface of tumor cells and FPR1 on the surface of DC are stably combined with the forming cells to promote the maturation of DC, and DC presents antigens to T cells, further killing tumor cells.

Anthracyclines can trigger exposure to CRT, as well as HSP70 and HSP90 expression, which involves translocation of these markers to the cell surface, and thus induces anti-cancer immune responses and HMGB1 release (Obeid et al., 2007; Fucikova et al., 2011). (Figure 2). HSP70 and HSP90 can be transferred to the plasma membrane of dying tumor cells, presented the tumor antigen to CD8+T cells. In addition, HSP70 can also induce NK cell activation and promote DC maturation, thus inducing tumor cell death (Tesniere et al., 2008). Further, there are reports that anthracycline treatment can improve the phagocytosis rate of acute lymphoblastic leukemia tumor cells by DCs *in vitro* (Fucikova et al., 2011). Some chemotherapeutic agents act by inducing endoplasmic reticulum (ER) stress, which activates the serine/threonine kinase, protein kinase R-like endoplasmic reticulum kinase (PERK) and phosphorylation of eukaryotic translation initiation factor 2A (EIF2A, also known as eIF2α) (Obeid et al., 2007). In recent years, some researchers have provided new insights into the fundamentals of ICD. Bezu et al., observed that anthracyclines can induce EIF2A phosphorylation without continuously triggering other manifestations of ER stress, where EIF2A phosphorylation is strongly associated surface exposure of CRT, a characteristic marker of ICD (Bezu et al., 2018). In addition, Vacchelli et al. reported that ICD induced by anthracyclines depends on the release of Annexin A1 (ANXA1) in cancer cells and formyl peptide receptor 1 (FPR1) during the late stage of DC-driven chemotaxis (Vacchelli et al., 2015).

Many anthracyclines can induce ICD, including, but not limited to, DOX, epirubicin, daunorubicin, idarubicin, and MTX (Fucikova et al., 2011; Garg et al., 2014). DOX, an anthracycline antibiotic, has been used to treat cancer for more than 40 years and is among the most effective anticancer drugs (D'Angelo et al., 2022). Although DOX has a wide range of biochemical effects on tumor cells, it indues significant cytotoxicity in many organs, particularly the heart; the underlying mechanism involves mitochondria damage, iron overload, and perturbed Ca^2+^ homeostasis, which lead to myocardial damage (Wu et al., 2022). Camilio et al. studied the potential of combined treatment of triple-negative breast cancer using the oncolytic peptide, LTX-315, together with DOX, and found that these two reagents can trigger anti-cancer immune responses, thus increasing T cell infiltration and limiting tumor growth (Camilio et al., 2019). Chemotherapy-induced ICD has proven beneficial immunostimulatory effects on tumor treatment. Loy et al. reported that neoadjuvant chemotherapy with DOX and CPA can increase numbers of tumor infiltrating lymphocytes, which is associated with favorable prognosis in patients with triple-negative breast cancer (Loi et al., 2016); however, there was no significant difference in adaptive immune response between animals treated with DOX liposomes alone and untreated control groups in experiments using the highly invasive 4T1 mouse breast cancer tumor model (Wu et al., 2020). Gao et al. found that a combination treatment with DOX and the small molecule IDO1 inhibitor, NLG919, significantly inhibited the growth of 4T1 murine breast cancer cells compared with single treatments, which only slightly limited the tumor growth rate (Gao et al., 2019). In addition, Zhuo et al. reported that a combination of low dose DOX and icariin acted synergistically to induce ICD, thereby improving the curative effects of ICD in hepatocellular carcinoma (Yu et al., 2020).

Although ICD-induced chemotherapy drugs bring new possibilities for tumor immunotherapy, the risk of side effects and systemic toxicity is still a big challenge in this field. In a clinical trial, the use of anthracycline for patients with ERBB2 (formerly HER2)-positive breast cancer in the presence of double ERBB2 blockade will increase the risk of febrile neutropenia and cardiotoxic effects (van der Voort et al., 2021). A major factor limiting the clinical application of anthracyclines is myocardial toxicity (Wu et al., 2022). Compared with traditional anthracycline drugs, anthracycline liposome preparations exhibit significantly reduced cardiotoxicity due to the influence of microvascular penetration (Henriksen, 2018). Deng et al. invented a liposome, PEG-FA-Lip, to deliver DOX, which can promote DC maturation and secretion of immune stimulating factors, effectively triggering T cell immune responses, and thus improving its therapeutic effect on solid tumors (Deng et al., 2019). Monk et al. reported that combining a Toll-like receptor 8 (TLR8) agonist with pegylated liposomal DOX significantly inhibited the growth of ovarian carcinoma in mice with a humanized immune system (Monk et al., 2017). Mastria et al. proved that chimeric polypeptide DOX (a nanoparticle DOX preparation) significantly enhanced anti-cancer immunity by stimulating CD8^+^ T cells and limiting tumor growth, metastasis, and spread (Mastria et al., 2018). Further, Xia et al. designed a double fluorescence imaging-guided programmed delivery system including DOX and cytosine-phosphate-guanine nanoparticles, which produced good anti-tumor therapeutic effects by regulating the tumor microenvironment and promoting CD4^+^ and CD8^+^ T cell infiltration (Dong et al., 2020). In addition, because DOX-induced cardiotoxicity is related to oxidative stress, according to this mechanism, several cardioprotective drugs including dexrazoxane, statins and coenzyme Q10 have been proved to be effective in the mouse model (Trajković et al., 2007; Seicean et al., 2012; Chen et al., 2017). However, due to the lack of large-scale clinical trials, these agents anthracycline should be further studied to reduce the toxicity of anthracycline to the heart. Further, daunorubicin was reported to trigger strong upregulation of CRT on the surface of primary human CD34 acute myeloid leukemia (AML) cells, inducing ICD (Aurelius et al., 2019). Idarubicin is a 4-demethoxyanthracycline analog of daunorubicin, which can be used to treat acute myelogenous leukemia (Coombs et al., 2016). According to data from a retrospective study, high-dose cytarabine plus idarubicin consolidation therapy had strong anti-leukemia effects in patients with fms-like tyrosine kinase (FLT3) wild-type and mutant AML that carried an nucleophosmin 1 (NPM1) mutation (Borlenghi et al., 2022). During a search for effective drugs against CD105 glioblastoma (GBM), Li et al. determined that idarubicin has strong toxic effects against CD105 GBM cells (Li et al., 2022).

Epirubicin, a type of anthracycline, interferes with DNA and RNA synthesis, and can be combined with other chemotherapeutic drugs to treat various malignant tumors, including postoperative lung cancer and breast cancer (Cameron et al., 2017; Annic et al., 2022; Schneeweiss et al., 2022). In addition, Li et al. engineered a self-amplified biomimetic nanosystem, in which epirubicin, glucose oxidase, and hemin are encapsulated within the core of ZIF-8 nanoparticles, which significantly induced ICD to promote DC maturation and CTL infiltration into tumor lesions (Li et al., 2023).

MTX, a broad-spectrum anti-tumor anthracycline, has also been shown to induce ICD in melanoma, prostate cancer, osteosarcoma, and mouse colon cancer cells through an eIF2α phosphorylation-dependent mechanism, leading to anti-cancer immune responses (Bezu et al., 2018; Giglio et al., 2018; Qin et al., 2019; Li et al., 2020) (Figure 2).

### 2.2 Platinum drugs

Chemotherapy combined with oxaliplatin (OXA) remains among the main treatments for most patients with CRC (colorectal cancer) (McQuade et al., 2017). Unlike cisplatin, OXA alone or combined with antimetabolite agents (trifluridine/tipiracil) can stimulate CRT to transfer from the ER to the cell surface in mouse colon cancer cells, thus inducing immunogenic death (Limagne et al., 2019). To improve anti-tumor efficacy, resistance of CRC cells to OXA can be avoided by inhibiting the serine/threonine kinase, ATR (Combès et al., 2019). Further, in mouse hepatocellular carcinoma cell lines, oxaliplatin can significantly increase the levels of ICD-related markers in the supernatant, and recruit immune cells to the tumor by inducing T cell accumulation, thus effectively inhibiting tumor growth. Similarly, levels of HMGB1 and ATP are also significantly upregulated in human hepatocellular carcinoma cells (Zhu et al., 2020). Comparable results were reported in murine glioma cells (KR158), murine lung carcinoma (LLC), murine mammary adenocarcinoma (TSA), and human (Panc-1) and murine (Pan02) pancreatic tumor cell lines (Golden et al., 2014; Zhao X. et al., 2016; Roberts et al., 2018; Sun et al., 2019).

Groza et al. studied the auxiliary role of “bacterial ghosts” (i.e., empty envelopes of Gram-negative bacteria) in OXA chemotherapy, and combined them with OXA to trigger anti-tumor T cell responses against CT26 murine colon cancer cells and establish long-term immune memory (Groza et al., 2018). Kanekiyo et al. conducted therapy based on combination of five HLA-A*24:02-restricted peptide vaccines with OXA, which caused peptide-specific IgG responses and improved overall survival (OS) of patients with CRC (Kanekiyo et al., 2018). In the latest research, liposomal OXA prodrugs loaded with metformin were demonstrated to enhance cancer immunotherapy, the alleviation of tumor hypoxia by metformin helps OXA induce ICD in mouse colorectal tumor cells (Song et al., 2022). Zhou et al. reported a type of prodrug vesicle, which integrated an OXA prodrug and a PEGylated photosensitizer, and further improved the curative effect of anticancer immunotherapy by inducing ICD and blocking CD47, to promote antigen presentation by DCs in mouse colorectal tumors, breast tumors, and melanoma cells (Zhou et al., 2019). Guo et al. developed a nanoparticle preparation containing OXA derivatives and folinic acid to induce ICD and inhibit tumor growth; use of the preparation in combination with nano-preparations containing active metabolites of 5-Fu synergistically increased its curative effect on CRC and hepatocellular carcinoma in mouse models, due to reactive oxygen species generation (Guo et al., 2021). Further, Wang et al. found that, when combined with a low dose of OXA, thiostrepton, an antibiotic produced by *Streptomyces*, can enhance anti-cancer immunogenicity by promoting the release of ATP and HMGB1, as well as CRT exposure, in mouse fibrosarcoma cells (Wang Y. et al., 2020). (Figure 2).

Notably, although cisplatin and OXA show considerable structural overlap, cisplatin cannot induce genuine ICD. Cis-diamminedichloroplatinum (better known as cisplatin or CDDP) is a platinum drug that is widely used for treatment of malignant tumors and exhibits significant therapeutic effects against testicular germ cell, colorectal, ovarian, bladder, lung, and head and neck cancers (Galanski, 2006; Ghosh, 2019); however, it differs from OXA in the levels of CRT exposure it induces, and cannot activate PERK-dependent eIF2α phosphorylation (Martins et al., 2011). In experiments where CDDP and OXA were each applied to mouse hepatoma cells, there was no significant difference between CDDP and the control group, demonstrating that ICD was not effectively induced (Zhu et al., 2020). Another study reported that treatment of mouse LLC lung cancer cells with CDDP (2.5 μM) could induce CRT exposure and ATP release, although it could not induce HMGB1 release (Aranda et al., 2015). Further, Sun et al. reported that no ICD induction was found after treatment of LLC cells with CDDP (20 μM) for 24 h (Sun et al., 2019); however, in some specific cases, CDDP combined with ischemia and reperfusion injury can lead to ICD in murine LLC cells (Zhang et al., 2022). Further, there are reports that CDDP can induce CXCL10 expression in melanoma cells, compared with untreated controls (Luo et al., 2019). In addition to research on the effects of these two drugs in inducing ICD, there is evidence that carboplatin can induce the release of HMGB1 and CRT in Colon26 and MC38 cells (Schaer et al., 2019).

The toxicity of OXA is still one of the limitations of clinical application. Although OXA is less ototoxic and nephrotoxic than cisplatin, it still causes various adverse effects including neurotoxicity which is difficult to prevent. (Sałat, 2020). According to the current results of clinical trials, nodrug can be a gold standard to prevent the neurotoxicity of OXA and the preventive measure depends on the dose adjustment of the OXA (Poupon et al., 2015). In addition, liposome encapsulation is also an effective strategy to reduce the damage to normal tissues. In addition to antibody-drug conjugate, the peptide-drug conjugates can improve efficacy and reduce side effects for cancer treatment in cell and animal studies (Alas et al., 2021).

### 2.3 Proteasome inhibitors

**FIGURE 3:**
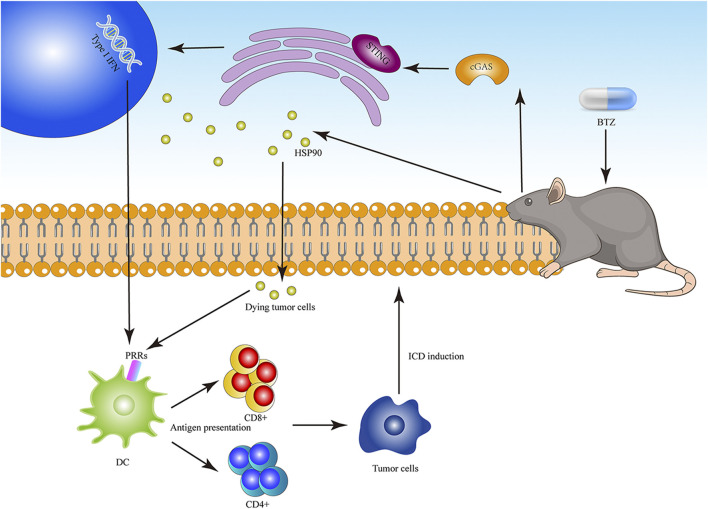
The diagram of BTZ to induce ICD. BTZ can significantly induce the expression of HSP90 on the cell surface and mediate the maturation of DC. BTZ triggers ICD by activating cGAS/STING pathway and producing type I IFN.

BTZ is a specific inhibitor of the 26S proteasome subunit, which induces human tumor cell apoptosis through various mechanisms, exhibits good clinical activity in multiple myeloma, lymphoma, breast cancer, lung cancer, and CRC (Liu et al., 2021), and can induce ICD. In myeloma (U266 and CAG), breast cancer (MCF-7), and mantle cell lymphoma (NCEB-1) cell lines, BTZ significantly induced cell surface expression of HSP90 and mediated DC maturation, thus enhancing tumor cell immunogenicity (Spisek et al., 2007). BTZ triggers ICD in multiple myeloma cells by activating the cGAS/STING pathway and producing type I IFN, which can be significantly enhanced by STING agonists (Gulla et al., 2021). In recent years, researchers have explored numerous strategies to improve BTZ-mediated ICD (Figure 3). In research related to nano-drugs, smart pH-responsive polyhydrazine/BTZ nanoparticles were more effective in inducing ICD of 4T1 cells and inhibiting lung metastasis than BTZ alone (Wang et al., 2022). BTZ combined with radiotherapy can enhance colon cancer cell sensitivity to apoptosis, which can significantly increase the killing effect of tumor-specific CD8⁺ T cells on colon cancer cells, thus effectively inducing anti-tumor immunity (Cacan et al., 2015). Recent research demonstrated that cell surface translocation of CRT was enhanced by a combination of BTZ and geranylgeranyl diphosphate synthase inhibitor, leading to enhanced immunogenicity (Haney et al., 2022). Carfilzomib is also a proteasome inhibitor and CRT exposure was detected in human multiple myeloma cells (MM.1S, U266, H929) treated with carfilzomib, while immunogenicity was enhanced when carfilzomib was combined with chloroquine (Jarauta et al., 2016). In the aspect of side effect, compared with other chemotherapy agents, BTZ has safer efficacy and controllable toxicity. Subcutaneous administration of bortezomib, as an alternative to intravenous administration, significantly reduced the probability of peripheral neuropathy while maintaining efficacy (Tan et al., 2019).

### 2.4 Alkylating agents

Cyclophosphamide (CPA) is a drug used for a wide range of cancer treatments, and has remarkable curative effects on lymphoma and solid tumors, including breast cancer, ovarian cancer, and bone and soft tissue sarcoma (Emadi et al., 2009). Further, the therapeutic effects of CPA can be achieved by stimulating immune cells to generate an immune response. In mouse thymoma cells (EG7), CPA can induce immunogenic tumor cell apoptosis and release of large amounts of HMGB1 (Schiavoni et al., 2011). A circulating low-dose CPA regimen restored peripheral T cell proliferation and innate killing activities by inhibiting human CD4^+^ CD25^+^regulatory T cells (Ghiringhelli et al., 2007). In addition, metronomic CPA treatment for subcutaneous growth of GL261 GBM tumors activates strong innate anti-tumor immunity in mice, thus effectively inducing immune-based tumor regression (Wu and Waxman, 2014). Similarly, metronomic CPA treatment improves the survival rate of model mice with subcutaneous GL261 GBM tumors by influencing immune function (Ferrer-Font et al., 2017). Nayagom et al., reported sensitization of tumor cells to anticancer agents by transfer of suicide genes; mesenchymal stem cells expressing suicide genes metabolize CPA into toxic metabolites, further induce ICD and DAMPs release, and significantly enhance tumor immunogenicity (Nayagom et al., 2019). The phenomenon whereby CPA metabolites induce ICD provides potential for improved tumor eradication, as well as additional possibilities for anti-tumor immunotherapy.

### 2.5 Paclitaxel

Paclitaxel (PTX) is an important and effective anti-tumor drug, which can quickly bind and stabilize microtubules (Yang and Horwitz, 2017). A liposomal PTX formulation has been used to treat ovarian cancer, breast cancer, and non-small cell lung cancer (NSCLC) (Gilabert-Oriol et al., 2018). David et al. found that the tumor tissues from mice treated with PLX3397/PTX enhanced anti-tumor immunity, blocked tumor-associated macrophage infiltration, and were beneficial to tumor inhibition mediated by CD8^+^ T cells (DeNardo et al., 2011).

Low-dose nano-PTX is proven to promote T cell infiltration into CT26 tumors (Yang et al., 2020). PTX has been proposed as an effective ICD inducer based on research into mouse breast cancer (4T1), CRC (CT26 and MC38), lung cancer (LL/2), and ovarian cancer (ID8 and ID8F3) cells, as well as human CRC cells (HCT116), where it causes the exposure of CRT and ERp57 in tumor cells, accompanied by ATP and HMGB1 release (Lau et al., 2020; Yang et al., 2020). In addition, combined nano PTX and programmed cell death protein 1 (PD-1) antibody treatment of colon tumors in model mice was significantly more effective than single drug treatment (Yang et al., 2020). Song et al. developed a nanogel encapsulating PTX to deliver interleukin −2 (IL-2), which significantly induced CRT exposure and enhanced anti-tumor activity (Song et al., 2017).

Nano-preparations may also be a good choice to reduce side effects. Nab-paclitaxel, a nano-preparation of paclitaxel, reduces the frequency of neuropathy, anemia, pain and diarrhea in patients with metastatic breast cancer (Mahtani et al., 2018).

### 2.6 Lasted finalized clinical studies

Following Vanmeerbeek’s trial watch in 2020 (Vanmeerbeek et al., 2020), many latest clinical trials have evaluated the real efficacy of various ICD-induced drugs. We summarized several clinical trials related to the efficacy of drugs induced ICD (Table 2). Sawaki and collaborators reported the results of a randomized controlled trial enrolling 275 older patients202 with HER2-positive early breast cancer. In this context, the disease-free survival of trastuzumab plus chemotherapy drugs including PTX, Docetaxel, Epirubicin, DOX and CPA (93.8%) was superior to trastuzumab alone (89.5%) (Sawaki et al., 2020). Yamaguchi et al., treated 90 patients with gastric/gastroesophageal junction cancer with pembrolizumab in combination with oxaliplatin or cisplatin. In the context of this phase IIb study, it provided strong evidence for the efficacy of ICD-induced chemotherapy drug combined with ICBs. However, there is no difference in two kinds of platinum agents (Yamaguchi et al., 2022). Lee et al., combined pegylated liposomal doxorubicin (PLD) and pembrolizumab in 200 patients with ovarian cancer, found that the ORR and median PFS of combination therapy was better than historical therapy of pembrolizumab alone (Lee et al., 2020). In the context of a phase IIb study of Wildiers et al., combined pertuzumab and trastuzumab with metronomic CPA chemotherapy improve the therapeutic effect on patients with HER2-positive metastatic breast cancer. The PFS of metronomic oral CPA plus trastuzumab and pertuzumab (28.7%) was superior to trastuzumab and pertuzumab (18.7%) (Wildiers et al., 2022). Zsiros et al. treated patiens with ovarian cancer. The combination of cyclophosphamide, pabolizumab and bevacizumab showed good clinical benefits (median PFS = 10.0) in the phase 2 clinical trial (Zsiros et al., 2021).

**TABLE 2:** The recent clinical studies to evaluate the combination of ICD-induced chemotherapy and immunotherapy.

Drug	Indications	Phase	Notes	Ref
Multimodal chemotherapy	Breast cancer	—	Combined with trastuzumab	Sawaki et al. (2020)
OXA	Gastric/gastroesophageal junction	II	Combined with pembrolizumab	Yamaguchi et al. (2022)
OXA	Gallbladder cancer	—	Combined with apatinib and gemcitabine	Qu et al. (2022)
CPA	Breast cancer	II	Combined with pertuzumab and trastuzumab	Wildiers et al. (2022)
CPA	Ovarian cancer	II	Combined with pembrolizumab and bevacizumab	Zsiros et al. (2021)
PLD	Ovarian cancer	II	Combined with pembrolizumab	Lee et al. (2020)

Other clinical trials focused on biomarkers of immune in patients treated with ICD-induced chemotherapeutics. Qu et al. mentioned that the treatment of gemcitabine and oxaliplatin combined with apatinib in gallbladder cancer will significantly increase the level of humoral immune cells, and significantly decrease the levels of sIL-2R, and sICAM-1 to effectively control the progress of the disease by improving the immune function (Qu et al., 2022). According to the current evidence, breast cancer and colorectal cancer may be indications that ICD-induced chemotherapy drugs can be combined with immune drugs to obtain good clinical activity (Vanmeerbeek et al., 2020). In summary, ICD-induced chemotherapy may provide benefits for patients receiving immunotherapy.

## 3 ICD in targeted therapy

### 3.1 Targeting ICD in immunotherapy

Common ways to induce cell death include necroptosis, apoptosis, and pyroptosis. Apoptosis can be divided into intrinsic and extrinsic forms (Bock and Tait, 2020). Among them, mitochondrial outer membrane permeabilization (MOMP) plays a key role in intrinsic apoptosis by activating NF-κB (Bock and Tait, 2020). Caspase proteases can also inhibit IFN reaction and indirectly inactivate HMGB1 in damps to keep apoptosis immunologically silent, thus affecting the process of apoptosis (Kazama et al., 2008; Rongvaux et al., 2014; Ning et al., 2019). Therefore, ICD can be strongly induced by type I IFN responses, which are mediated by blocking caspase and MOMP(Rongvaux et al., 2014; Giampazolias et al., 2017). Immunogenic chemotherapeutic drugs that can induce ICD play an anti-tumor role by mediating the release of damps in addition to inducing apoptosis (Messmer et al., 2019). Besides, anti-PD-L1 and emricasan (a caspase inhibitor) combined with radiation can induce anti-tumor effect for more effective immunotherapy (Han et al., 2020). The immunogenic mechanism of necroptosis is still controversial. Annelise et al., demonstrated that the anti-tumor immunity of necroptosis depended on the induction of NF-κB mediated by protein kinases RIPK1 and RIPK3, while Tania et al., believed that the immunogenicity was related to the release of damps, rather than NF-κB (Aaes et al., 2016; Snyder et al., 2019). Necroptosis cancer cells have been proved to be effective inducers of anti-tumor immune response and used for tumor vaccination. The targeting necroptosis brings new possibilities for cancer treatment (Aaes et al., 2016). Similarly, pyroptosis induced by gasdermin D (GSDMD) is also an important form of cell death related to the mechanism of chemotherapy drugs killing tumor cells (Wang et al., 2017). The recovery of GSDME expression in tumor cells is helpful to enhance the function of immune cells to inhibit tumor growth (Wang et al., 2017). The activation of pyroptosis can trigger strong anti-tumor immunity which is synergistic with anti-PD1 immune checkpoint blockade (Wang Q. et al., 2020).

### 3.2 ICD induced by targeted drugs

In fact, anti-tumor immune responses related to anti-cancer drug-induced therapy are not limited to traditional chemotherapy drugs. Although there is no clear evidence of ICD induced by tyrosine kinase in the related research of ICD induced by targeted drugs. Some evidence showed that anti-EGFR specific antibody (7A7) can cause significant tumor-specific CTL response, and their clinical efficacy is related to ICD induction (Garrido et al., 2011). In the study of D122 mice lung cancer cells, 7A7, like anthracycline-induced ICD, can induce CRT and ERp12 on plasma membrane to be exposed to the cell surface, and cause the early phosphorylation of eIF6α. Dying D122 cells treated with 7A7 also made a major contribution to stimulate DC maturation, and increased the infiltration of CD4+T cells and CD8+T cells (Garrido et al., 2007). Anti-tumor immune responses also extend to numerous targeted drugs (Galluzzi et al., 2015; Petroni et al., 2021) (Table 3), such as cetuximab, which is used for treatment of patients with metastatic colorectal cancer (mCRC) (Modest et al., 2019). Research using human and mouse CRC cell lines demonstrated that cetuximab triggered an ER stress response and promoted DC phagocytosis. The immunogenicity of a cetuximab-treated mouse CRC cell line (CT26) expressing human EGFR (hEGFR-CT26) increased and the tumor cells induced an effective anti-tumor response (Pozzi et al., 2016). Ibrutinib is another targeted drug with significant anti-tumor effects against chronic lymphocytic leukemia, which has shown sustained benefits for patients in clinical studies (Barr et al., 2022). In experiments using mouse lymphoma cell lines (H11, A20, and BL3750), ibrutinib enhanced the anti-tumor immune response induced by intratumoral injection of a toll like receptor 9 (TLR9) ligand, and promoted T cell-dependent tumor regression (Sagiv-Barfi et al., 2015). Liu et al. detected CRT exposure and ATP and HMGB1 release in several human cancer cell lines (U2OS, HeLa, HCT-116) and mouse fibrosarcoma cells (MCA205) following treatment with various tyrosine kinase inhibitors, including: (R)-crizotinib, foretinib, canertinib, lestaurtinib, and ceritinib. These characteristics are similar to those of the anthracycline ICD inducer, MTX, and suggest that crizotinib has potential to act as an ICD inducer. In addition, they found that combination treatment of NSCLC with cisplatin and crizotinib induced ICD *in vivo* (Liu et al., 2019). Further, Petrazzuolo et al. found that crizotinib and ceritinib induced anaplastic lymphoma kinase (ALK)-dependent ICD in anaplastic large cell lymphoma (Petrazzuolo et al., 2021). Cyclin-dependent kinases 4 and 6 (CDK4/6) are key molecules involved in cell cycle regulation, which are closely associated with the occurrence and development of malignant tumors (Fassl et al., 2022). CDK4/6 inhibitors exhibit remarkable activity against several solid tumors, including breast cancer and NSCLC (Finn et al., 2016; Patnaik et al., 2016). There is increasing evidence that CDK4/CDK6 inhibitors can both inhibit malignant cell proliferation and mediate their broader regulation, including through immune stimulation (Petroni et al., 2020). Goel et al. proved that CDK4/6 inhibitors (abemaciclib, palbociclib, and lapatinib) can increase tumor cell antigen presentation ability, promote CTL-mediated tumor cell clearance, and enhance the immunogenicity of malignant cells by overcoming immune evasion in a mouse breast cancer model (Goel et al., 2017).

**TABLE 3:** The targeted drugs-induced ICD.

Targeted drug	Cancer type	Mechanism	ICD effect	Ref
Anti-EGFR monoclonal antibody (cetuximab)	Metastatic colorectal cancer (mCRC)	Trigger ER stress response and promote DC phagocytosis	Induce effective anti-tumor response	Pozzi et al. (2016)
Bruton’s tyrosine kinase Inhibitor (ibrutinib)	Chronic lymphocytic leukemia (CLL)	Promote T cell-dependent tumor regression	The combination of ibrutinib and an agonist for the toll-like receptor 9 enhances anti-tumor immune response	Sagiv-Barfi et al. (2015)
Tyrosine kinase inhibitors ((R)-crizotinib, foretinib, canertinib, lestourtinib and ceritinib)	NSCLC	Trigger several markers of ICD and induce phosphorylation of eIF2α	Increase the infiltration of tumor T cells and cause anti-cancer immune response	Liu et al. (2019)
Tyrosine kinase inhibitors (crizotinib and ceritinib)	Anaplastic large cell lymphoma (ALCL)	Inhibit anaplastic lymphoma kinase	Induce immune response and slow down the growth of ALCL cells	Petrazzuolo et al. (2021)
CDK4/CDK6 inhibitors (abemaciclib, palbociclib and lapatinib)	Breast cancer	Enhance antigen presentation and stimulate cytotoxic T cells	Increase tumor immunogenicity and induce tumor regression	Goel et al. (2017)

## 4 Conclusion and perspectives

Cancer chemoimmunotherapy is among the most effective combined strategies against tumor cells. Here, we reviewed the mechanisms of ICD induction by chemotherapy and targeted drugs, and the performance of various drugs in tumor models. Many ICD-induced chemotherapy regimens have been approved for widespread use in patients with cancer, although most use is based on empirical evidence (Hanoteau and Moser, 2016; Nikanjam et al., 2017; Zhang et al., 2017). Although some medical therapies based on ICD have made considerable progress, their clinical application continues to face many challenges. Side effects, systemic toxicity risk, and unpredictable curative effects of anti-tumor drugs are all problems that need to be solved. In addition, determining dosage and treatment plans that mediate maximal immunostimulation is an ongoing major challenge (Zhao X.-Y. et al., 2016; Wu and Waxman, 2018). Therefore, how to translate the results of basic research into chemotherapy-induced ICD into clinical practice remains a significant obstacle. The hallmarks of ICD (CRT exposure on the cell surface, ATP secretion, and HMGB1 release) have been analyzed as biomarkers to predict the prognosis and survival of patients with cancer with the aim of future clinical application (Galluzzi et al., 2015). Similarly, the mechanisms driving ICD have been used to produce DC-based tumor vaccines, and many clinical trials have been completed (Wculek et al., 2020). In summary, research into the mechanisms underlying ICD is expected to stimulate the discovery of new immunogenic anticancer drugs and provide a solid foundation for the development of more effective methods of cancer treatment.

## Figures and Tables

**Table:** 

**ICD**	immunogenic cell death
**DOX**	doxorubicin
**OXA**	oxaliplatin
**CPA**	cyclophosphamide
**BTZ**	bortezomib
**PTX**	paclitaxel
**ICD**	immunogenic cell death
**DAMPs**	damage-related molecular patterns
**HMGB1**	high mobility group box 1
**CRT**	calreticulin
**ATP**	adenosine triphosphate
**HSP**	heat shock protein
**MTX**	mitoxantrone
**ER**	endoplasmic reticulum
**P2RY2**	purinergic receptor P2Y2
**IL-1β**	interleukin 1 β
**IL-18**	interleukin-18
**DC**	dendritic cells
**ELISA**	enzyme-linked immunosorbent assay
**TLR4**	toll-like receptors 4
**APCs**	antigen presenting cells
**CTLs**	cytotoxic T lymphocytes
**IL-17**	interleukin-17
**FasL**	Fas ligand
**ANXA1**	annexin A1
**FPR1**	formyl peptide receptor 1
**IFN**	interferon
**CXCL1**	chemokine (C-X-C motif) ligand 1 protein
**CCL2**	C-C motif ligand 2
**CXCL10**	chemokine (C-X-C motif) ligand 10 protein
**PERK**	protein kinase R-like endoplasmic reticulum kinase
**EIF2A**	translation initiation factor 2A
**TILs**	tumor infiltrating lymphocytes
**TLR8**	tolllike receptor 8
**CpG**	cytisine-phosphate-guanine
**FLT3**	fms-like tyrosine kinase
**AML**	acute myeloid leukemia
**NPM1**	nucleophosmin 1
**GBM**	glioblastoma
**OS**	overall survival
**CDDP**	Cis-diamminedichloroplatinum
**LLC**	lung cancer cells
**NSCLC**	non-small cell lung carcinoma
**PD-1**	programmed cell death protein 1
**IL-2**	interleukin -2
**mCRC**	metastatic colorectal cancer
**CRC**	colorectal cancer
**EGFR**	epidermal growth factor receptor
**CLL**	chronic lymphocytic leukemia
**TLR9**	toll Like Receptor 9
**ALK**	anaplastic lymphoma kinase
**CDK4/6**	cyclin-dependent kinases 4 and 6
**ICD**	immunogenic cell death
**DOX**	doxorubicin

## References

[B1] Aaes T. L. Kaczmarek A. Delvaeye T. De Craene B. De Koker S. Heyndrickx L. (2016). Vaccination with necroptotic cancer cells induces efficient anti-tumor immunity. Cell. Rep. 15, 274–287. 10.1016/j.celrep.2016.03.037 27050509

[B2] Alas M. Saghaeidehkordi A. Kaur K. (2021). Peptide-drug conjugates with different linkers for cancer therapy. J. Med. Chem. 64, 216–232. 10.1021/acs.jmedchem.0c01530 33382619 PMC8610607

[B3] Annic J. Babey H. Corre R. Descourt R. Quéré G. Renaud E. (2022). Real-life second-line epirubicin-paclitaxel regimen as treatment of relapsed small-cell lung cancer: EpiTax study. Cancer Med. 12, 2658–2665. 10.1002/cam4.5143 36000584 PMC9939142

[B4] Apetoh L. Ghiringhelli F. Tesniere A. Obeid M. Ortiz C. Criollo A. (2007). Toll-like receptor 4-dependent contribution of the immune system to anticancer chemotherapy and radiotherapy. Nat. Med. 13, 1050–1059. 10.1038/nm1622 17704786

[B5] Aranda F. Bloy N. Pesquet J. Petit B. Chaba K. Sauvat A. (2015). Immune-dependent antineoplastic effects of cisplatin plus pyridoxine in non-small-cell lung cancer. Oncogene 34, 3053–3062. 10.1038/onc.2014.234 25065595

[B6] Aurelius J. Möllgård L. Kiffin R. Ewald Sander F. Nilsson S. Thorén F. B. (2019). Anthracycline-based consolidation may determine outcome of post-consolidation immunotherapy in AML. Leukemia Lymphoma 60, 2771–2778. 10.1080/10428194.2019.1599110 30991860

[B7] Barr P. M. Owen C. Robak T. Tedeschi A. Bairey O. Burger J. A. (2022). Up to 8-year follow-up from RESONATE-2: First-line ibrutinib treatment for patients with chronic lymphocytic leukemia. Blood Adv. 6, 3440–3450. 10.1182/bloodadvances.2021006434 35377947 PMC9198904

[B8] Bell C. W. Jiang W. Reich C. F. 3rd Pisetsky D. S. (2006). The extracellular release of HMGB1 during apoptotic cell death. Am. J. Physiol. Cell. Physiol. 291, C1318–C1325. 10.1152/ajpcell.00616.2005 16855214

[B9] Bezu L. Sauvat A. Humeau J. Gomes-Da-Silva L. C. Iribarren K. Forveille S. (2018). eIF2α phosphorylation is pathognomonic for immunogenic cell death. Cell. Death Differ. 25, 1375–1393. 10.1038/s41418-017-0044-9 29358668 PMC6113215

[B10] Boada-Romero E. Martinez J. Heckmann B. L. Green D. R. (2020). The clearance of dead cells by efferocytosis. Nat. Rev. Mol. Cell. Biol. 21, 398–414. 10.1038/s41580-020-0232-1 32251387 PMC7392086

[B11] Bock F. J. Tait S. W. G. (2020). Mitochondria as multifaceted regulators of cell death. Nat. Rev. Mol. Cell. Biol. 21, 85–100. 10.1038/s41580-019-0173-8 31636403

[B12] Borlenghi E. Cattaneo C. Bertoli D. Cerqui E. Archetti S. Passi A. (2022). Prognostic relevance of *NPM1* and *FLT3* mutations in acute myeloid leukaemia, longterm follow-up-A single center experience. Cancers 14 (19):4716. 10.3390/cancers14194716 36230640 PMC9562865

[B13] Brown M. C. Holl E. K. Boczkowski D. Dobrikova E. Mosaheb M. Chandramohan V. (2017). Cancer immunotherapy with recombinant poliovirus induces IFN-dominant activation of dendritic cells and tumor antigen-specific CTLs. Sci. Transl. Med. 9, eaan4220. 10.1126/scitranslmed.aan4220 28931654 PMC6034685

[B14] Cacan E. Spring A. M. Kumari A. Greer S. F. Garnett-Benson C. (2015). Combination treatment with sublethal ionizing radiation and the proteasome inhibitor, bortezomib, enhances death-receptor mediated apoptosis and anti-tumor immune attack. Int. J. Mol. Sci. 16, 30405–30421. 10.3390/ijms161226238 26703577 PMC4691179

[B15] Cameron D. Morden J. P. Canney P. Velikova G. Coleman R. Bartlett J. (2017). Accelerated versus standard epirubicin followed by cyclophosphamide, methotrexate, and fluorouracil or capecitabine as adjuvant therapy for breast cancer in the randomised UK TACT2 trial (CRUK/05/19): A multicentre, phase 3, open-label, randomised, controlled trial. Lancet. Oncol. 18, 929–945. 10.1016/S1470-2045(17)30404-7 28600210 PMC5489700

[B16] Camilio K. A. Wang M.-Y. Mauseth B. Waagene S. Kvalheim G. Rekdal Ø. (2019). Combining the oncolytic peptide LTX-315 with doxorubicin demonstrates therapeutic potential in a triple-negative breast cancer model. Breast Cancer Res. BCR 21, 9. 10.1186/s13058-018-1092-x 30670061 PMC6343247

[B17] Cauwels A. Van Lint S. Garcin G. Bultinck J. Paul F. Gerlo S. (2018). A safe and highly efficient tumor-targeted type I interferon immunotherapy depends on the tumor microenvironment. Oncoimmunology 7, e1398876. 10.1080/2162402X.2017.1398876 29399401 PMC5790344

[B18] Chao M. P. Jaiswal S. Weissman-Tsukamoto R. Alizadeh A. A. Gentles A. J. Volkmer J. (2010). Calreticulin is the dominant pro-phagocytic signal on multiple human cancers and is counterbalanced by CD47. Sci. Transl. Med. 2, 63ra94. 10.1126/scitranslmed.3001375.ra94 PMC4126904 21178137

[B19] Chen P.-Y. Hou C.-W. Shibu M. A. Day C. H. Pai P. Liu Z.-R. (2017). Protective effect of Co-enzyme Q10 on doxorubicin-induced cardiomyopathy of rat hearts. Environ. Toxicol. 32, 679–689. 10.1002/tox.22270 27087047

[B20] Choi J. Shim M. K. Yang S. Hwang H. S. Cho H. Kim J. (2021). Visible-light-triggered prodrug nanoparticles combine chemotherapy and photodynamic therapy to potentiate checkpoint blockade cancer immunotherapy. ACS Nano 15, 12086–12098. 10.1021/acsnano.1c03416 34165970

[B21] Combès E. Andrade A. F. Tosi D. Michaud H.-A. Coquel F. Garambois V. (2019). Inhibition of ataxia-telangiectasia mutated and RAD3-related (*ATR*) overcomes oxaliplatin resistance and promotes antitumor immunity in colorectal cancer. Cancer Res. 79, 2933–2946. 10.1158/0008-5472.CAN-18-2807 30987998

[B22] Coombs C. C. Tallman M. S. Levine R. L. (2016). Molecular therapy for acute myeloid leukaemia. Nat. Rev. Clin. Oncol. 13, 305–318. 10.1038/nrclinonc.2015.210 26620272 PMC5525060

[B23] D'angelo N. A. Noronha M. A. Câmara M. C. C. Kurnik I. S. Feng C. Araujo V. H. S. (2022). Doxorubicin nanoformulations on therapy against cancer: An overview from the last 10 years. Biomater. Adv. 133, 112623. 10.1016/j.msec.2021.112623 35525766

[B24] Denardo D. G. Brennan D. J. Rexhepaj E. Ruffell B. Shiao S. L. Madden S. F. (2011). Leukocyte complexity predicts breast cancer survival and functionally regulates response to chemotherapy. Cancer Discov. 1, 54–67. 10.1158/2159-8274.CD-10-0028 22039576 PMC3203524

[B25] Deng C. Zhang Q. Jia M. Zhao J. Sun X. Gong T. (2019). Tumors and their microenvironment dual-targeting chemotherapy with local immune adjuvant therapy for effective antitumor immunity against breast cancer. Adv. Sci. (Weinheim, Baden-Wurttemberg, Ger. 6, 1801868. 10.1002/advs.201801868 PMC6425447 30937266

[B26] Dong X. Yang A. Bai Y. Kong D. Lv F. (2020). Dual fluorescence imaging-guided programmed delivery of doxorubicin and CpG nanoparticles to modulate tumor microenvironment for effective chemo-immunotherapy. Biomaterials 230, 119659. 10.1016/j.biomaterials.2019.119659 31831223

[B27] Emadi A. Jones R. J. Brodsky R. A. (2009). Cyclophosphamide and cancer: Golden anniversary. Nat. Rev. Clin. Oncol. 6, 638–647. 10.1038/nrclinonc.2009.146 19786984

[B28] Farhood B. Najafi M. Mortezaee K. (2019). CD8(+) cytotoxic T lymphocytes in cancer immunotherapy: A review. J. Cell. Physiol. 234, 8509–8521. 10.1002/jcp.27782 30520029

[B29] Fassl A. Geng Y. Sicinski P. (2022). CDK4 and CDK6 kinases: From basic science to cancer therapy. Science 375, eabc1495. 10.1126/science.abc1495 35025636 PMC9048628

[B30] Fend L. Yamazaki T. Remy C. Fahrner C. Gantzer M. Nourtier V. (2017). Immune checkpoint blockade, immunogenic chemotherapy or IFN-α blockade boost the local and abscopal effects of oncolytic virotherapy. Cancer Res. 77, 4146–4157. 10.1158/0008-5472.CAN-16-2165 28536278

[B31] Feng M. Jiang W. Kim B. Y. S. Zhang C. C. Fu Y.-X. Weissman I. L. (2019). Phagocytosis checkpoints as new targets for cancer immunotherapy. Nat. Rev. Cancer 19, 568–586. 10.1038/s41568-019-0183-z 31462760 PMC7002027

[B32] Ferrer-Font L. Arias-Ramos N. Lope-Piedrafita S. Julià-Sapé M. Pumarola M. Arús C. (2017). Metronomic treatment in immunocompetent preclinical GL261 glioblastoma: Effects of cyclophosphamide and temozolomide. NMR Biomed. 30 (9). 10.1002/nbm.3748 28570014

[B33] Finn R. S. Martin M. Rugo H. S. Jones S. Im S.-A. Gelmon K. (2016). Palbociclib and letrozole in advanced breast cancer. N. Engl. J. Med. 375, 1925–1936. 10.1056/NEJMoa1607303 27959613

[B34] Fucikova J. Kralikova P. Fialova A. Brtnicky T. Rob L. Bartunkova J. (2011). Human tumor cells killed by anthracyclines induce a tumor-specific immune response. Cancer Res. 71, 4821–4833. 10.1158/0008-5472.CAN-11-0950 21602432

[B35] Fucikova J. Spisek R. Kroemer G. Galluzzi L. (2021). Calreticulin and cancer. Cell. Res. 31, 5–16. 10.1038/s41422-020-0383-9 32733014 PMC7853084

[B36] Galanski M. (2006). Recent developments in the field of anticancer platinum complexes. Recent Pat. Anti-cancer Drug Discov. 1, 285–295. 10.2174/157489206777442287 18221042

[B37] Galluzzi L. Bravo-San Pedro J. M. Kepp O. Kroemer G. (2016a). Regulated cell death and adaptive stress responses. Cell. Mol. Life Sci. CMLS 73, 2405–2410. 10.1007/s00018-016-2209-y 27048813 PMC11108439

[B38] Galluzzi L. Buqué A. Kepp O. Zitvogel L. Kroemer G. (2017). Immunogenic cell death in cancer and infectious disease. Nat. Rev. Immunol. 17, 97–111. 10.1038/nri.2016.107 27748397

[B39] Galluzzi L. Buqué A. Kepp O. Zitvogel L. Kroemer G. (2015). Immunological effects of conventional chemotherapy and targeted anticancer agents. Cancer Cell. 28, 690–714. 10.1016/j.ccell.2015.10.012 26678337

[B40] Galluzzi L. Spranger S. Fuchs E. López-Soto A. (2019). WNT signaling in cancer immunosurveillance. Trends Cell. Biol. 29, 44–65. 10.1016/j.tcb.2018.08.005 30220580 PMC7001864

[B41] Galluzzi L. Vitale I. Warren S. Adjemian S. Agostinis P. Martinez A. B. (2020). Consensus guidelines for the definition, detection and interpretation of immunogenic cell death. J. Immunother. Cancer 8, e000337. 10.1136/jitc-2019-000337 32209603 PMC7064135

[B42] Galluzzi L. Zitvogel L. Kroemer G. (2016b). Immunological mechanisms underneath the efficacy of cancer therapy. Cancer Immunol. Res. 4, 895–902. 10.1158/2326-6066.CIR-16-0197 27803050

[B43] Gao J. Deng F. Jia W. (2019). Inhibition of indoleamine 2,3-dioxygenase enhances the therapeutic efficacy of immunogenic chemotherapeutics in breast cancer. J. Breast Cancer 22, 196–209. 10.4048/jbc.2019.22.e23 31281723 PMC6597411

[B44] Gardella S. Andrei C. Ferrera D. Lotti L. V. Torrisi M. R. Bianchi M. E. (2002). The nuclear protein HMGB1 is secreted by monocytes via a non-classical, vesicle-mediated secretory pathway. EMBO Rep. 3, 995–1001. 10.1093/embo-reports/kvf198 12231511 PMC1307617

[B45] Garg A. D. Martin S. Golab J. Agostinis P. (2014). Danger signalling during cancer cell death: Origins, plasticity and regulation. Cell. Death Differ. 21, 26–38. 10.1038/cdd.2013.48 23686135 PMC3858605

[B46] Garg A. D. More S. Rufo N. Mece O. Sassano M. L. Agostinis P. (2017a). Trial watch: Immunogenic cell death induction by anticancer chemotherapeutics. Oncoimmunology 6, e1386829. 10.1080/2162402X.2017.1386829 29209573 PMC5706600

[B47] Garg A. D. Nowis D. Golab J. Vandenabeele P. Krysko D. V. Agostinis P. (2010). Immunogenic cell death, DAMPs and anticancer therapeutics: An emerging amalgamation. Biochim. Biophys. Acta 1805, 53–71. 10.1016/j.bbcan.2009.08.003 19720113

[B48] Garg A. D. Vandenberk L. Fang S. Fasche T. Van Eygen S. Maes J. (2017b). Pathogen response-like recruitment and activation of neutrophils by sterile immunogenic dying cells drives neutrophil-mediated residual cell killing. Cell. Death Differ. 24, 832–843. 10.1038/cdd.2017.15 28234357 PMC5423108

[B49] Garg A. D. Vandenberk L. Koks C. Verschuere T. Boon L. Van Gool S. W. (2016). Dendritic cell vaccines based on immunogenic cell death elicit danger signals and T cell-driven rejection of high-grade glioma. Sci. Transl. Med. 8, 328. 10.1126/scitranslmed.aae0105.ra327 26936504

[B50] Garrido G. Lorenzano P. Sánchez B. Beausoleil I. Alonso D. F. Pérez R. (2007). T cells are crucial for the anti-metastatic effect of anti-epidermal growth factor receptor antibodies. Cancer Immunol. Immunother. CII 56, 1701–1710. 10.1007/s00262-007-0313-4 17415565 PMC11031102

[B51] Garrido G. Rabasa A. Sánchez B. López M. V. Blanco R. López A. (2011). Induction of immunogenic apoptosis by blockade of epidermal growth factor receptor activation with a specific antibody. J. Immunol. 187, 4954–4966. 10.4049/jimmunol.1003477 21984704

[B52] Ghiringhelli F. Menard C. Puig P. E. Ladoire S. Roux S. Martin F. (2007). Metronomic cyclophosphamide regimen selectively depletes CD4+CD25+ regulatory T cells and restores T and NK effector functions in end stage cancer patients. Cancer Immunol. Immunother. CII 56, 641–648. 10.1007/s00262-006-0225-8 16960692 PMC11030569

[B53] Ghosh S. (2019). Cisplatin: The first metal based anticancer drug. Bioorg. Chem. 88, 102925. 10.1016/j.bioorg.2019.102925 31003078

[B54] Giampazolias E. Zunino B. Dhayade S. Bock F. Cloix C. Cao K. (2017). Mitochondrial permeabilization engages NF-κB-dependent anti-tumour activity under caspase deficiency. Nat. Cell. Biol. 19, 1116–1129. 10.1038/ncb3596 28846096 PMC5624512

[B55] Giglio P. Gagliardi M. Tumino N. Antunes F. Smaili S. Cotella D. (2018). PKR and GCN2 stress kinases promote an ER stress-independent eIF2α phosphorylation responsible for calreticulin exposure in melanoma cells. Oncoimmunology 7, e1466765. 10.1080/2162402X.2018.1466765 30221067 PMC6136861

[B56] Gilabert-Oriol R. Ryan G. M. Leung A. W. Y. Firmino N. S. Bennewith K. L. Bally M. B. (2018). Liposomal formulations to modulate the tumour microenvironment and antitumour immune response. Int. J. Mol. Sci. 19, 2922. 10.3390/ijms19102922 30261606 PMC6213379

[B57] Goel S. Decristo M. J. Watt A. C. Brinjones H. Sceneay J. Li B. B. (2017). CDK4/6 inhibition triggers anti-tumour immunity. Nature 548, 471–475. 10.1038/nature23465 28813415 PMC5570667

[B58] Golden E. B. Frances D. Pellicciotta I. Demaria S. Helen Barcellos-Hoff M. Formenti S. C. (2014). Radiation fosters dose-dependent and chemotherapy-induced immunogenic cell death. Oncoimmunology 3, e28518. 10.4161/onci.28518 25071979 PMC4106151

[B59] Gomes-Da-Silva L. C. Zhao L. Bezu L. Zhou H. Sauvat A. Liu P. (2018). Photodynamic therapy with redaporfin targets the endoplasmic reticulum and Golgi apparatus. Embo J. 37, e98354. 10.15252/embj.201798354 29807932 PMC6028029

[B60] Groza D. Gehrig S. Kudela P. Holcmann M. Pirker C. Dinhof C. (2018). Bacterial ghosts as adjuvant to oxaliplatin chemotherapy in colorectal carcinomatosis. Oncoimmunology 7, e1424676. 10.1080/2162402X.2018.1424676 29721389 PMC5927527

[B61] Gulla A. Morelli E. Samur M. K. Botta C. Hideshima T. Bianchi G. (2021). Bortezomib induces anti-multiple myeloma immune response mediated by cGAS/STING pathway activation. Blood Cancer Discov. 2, 468–483. 10.1158/2643-3230.BCD-21-0047 34568832 PMC8462183

[B62] Guo J. Yu Z. Sun D. Zou Y. Liu Y. Huang L. (2021). Two nanoformulations induce reactive oxygen species and immunogenetic cell death for synergistic chemo-immunotherapy eradicating colorectal cancer and hepatocellular carcinoma. Mol. Cancer 20, 10. 10.1186/s12943-020-01297-0 33407548 PMC7786897

[B63] Han C. Liu Z. Zhang Y. Shen A. Dong C. Zhang A. (2020). Tumor cells suppress radiation-induced immunity by hijacking caspase 9 signaling. Nat. Immunol. 21, 546–554. 10.1038/s41590-020-0641-5 32231300

[B64] Haney S. L. Varney M. L. Williams J. T. Smith L. M. Talmon G. Holstein S. A. (2022). Geranylgeranyl diphosphate synthase inhibitor and proteasome inhibitor combination therapy in multiple myeloma. Exp. Hematol. Oncol. 11, 5. 10.1186/s40164-022-00261-6 35139925 PMC8827146

[B65] Hanoteau A. Moser M. (2016). Chemotherapy and immunotherapy: A close interplay to fight cancer? Oncoimmunology 5, e1190061. 10.1080/2162402X.2016.1190061 27622046 PMC5006929

[B66] Henriksen P. A. (2018). Anthracycline cardiotoxicity: An update on mechanisms, monitoring and prevention. Heart (British Card. Soc. 104, 971–977. 10.1136/heartjnl-2017-312103 29217634

[B67] Inoue H. Tani K. (2014). Multimodal immunogenic cancer cell death as a consequence of anticancer cytotoxic treatments. Cell. Death Differ. 21, 39–49. 10.1038/cdd.2013.84 23832118 PMC3857623

[B68] Jarauta V. Jaime P. Gonzalo O. De Miguel D. Ramírez-Labrada A. Martínez-Lostao L. (2016). Inhibition of autophagy with chloroquine potentiates carfilzomib-induced apoptosis in myeloma cells *in vitro* and *in vivo* . Cancer Lett. 382, 1–10. 10.1016/j.canlet.2016.08.019 27565383

[B69] Kanekiyo S. Hazama S. Takenouchi H. Nakajima M. Shindo Y. Matsui H. (2018). IgG response to MHC class I epitope peptides is a quantitative predictive biomarker in the early course of treatment of colorectal cancer using therapeutic peptides. Oncol. Rep. 39, 2385–2392. 10.3892/or.2018.6288 29498403

[B70] Kazama H. Ricci J.-E. Herndon J. M. Hoppe G. Green D. R. Ferguson T. A. (2008). Induction of immunological tolerance by apoptotic cells requires caspase-dependent oxidation of high-mobility group box-1 protein. Immunity 29, 21–32. 10.1016/j.immuni.2008.05.013 18631454 PMC2704496

[B71] Kepp O. Bezu L. Yamazaki T. Di Virgilio F. Smyth M. J. Kroemer G. (2021). ATP and cancer immunosurveillance. Embo J. 40, e108130. 10.15252/embj.2021108130 34121201 PMC8246257

[B72] Kepp O. Senovilla L. Vitale I. Vacchelli E. Adjemian S. Agostinis P. (2014). Consensus guidelines for the detection of immunogenic cell death. Oncoimmunology 3, e955691. 10.4161/21624011.2014.955691 25941621 PMC4292729

[B73] Kopecka J. Salaroglio I. C. Righi L. Libener R. Orecchia S. Grosso F. (2018). Loss of C/EBP-β LIP drives cisplatin resistance in malignant pleural mesothelioma. Lung Cancer 120, 34–45. 10.1016/j.lungcan.2018.03.022 29748013

[B74] Krysko D. V. Garg A. D. Kaczmarek A. Krysko O. Agostinis P. Vandenabeele P. (2012). Immunogenic cell death and DAMPs in cancer therapy. Nat. Rev. Cancer 12, 860–875. 10.1038/nrc3380 23151605

[B75] Lau T. S. Chan L. K. Y. Man G. C. W. Wong C. H. Lee J. H. S. Yim S. F. (2020). Paclitaxel induces immunogenic cell death in ovarian cancer via TLR4/IKK2/SNARE-dependent exocytosis. Cancer Immunol. Res. 8, 1099–1111. 10.1158/2326-6066.CIR-19-0616 32354736

[B76] Lee E. K. Xiong N. Cheng S.-C. Barry W. T. Penson R. T. Konstantinopoulos P. A. (2020). Combined pembrolizumab and pegylated liposomal doxorubicin in platinum resistant ovarian cancer: A phase 2 clinical trial. Gynecol. Oncol. 159, 72–78. 10.1016/j.ygyno.2020.07.028 32771276

[B77] Li C. Sun H. Wei W. Liu Q. Wang Y. Zhang Y. (2020). Mitoxantrone triggers immunogenic prostate cancer cell death via p53-dependent PERK expression. Cell. Oncol. Dordr. 43, 1099–1116. 10.1007/s13402-020-00544-2 32710433 PMC12990691

[B78] Li J. Ek F. Olsson R. Belting M. Bengzon J. (2022). Glioblastoma CD105^+^ cells define a SOX2^-^ cancer stem cell-like subpopulation in the pre-invasive niche. Acta Neuropathol. Commun. 10, 126. 10.1186/s40478-022-01422-8 36038950 PMC9426031

[B79] Li Z. Cai H. Li Z. Ren L. Ma X. Zhu H. (2023). A tumor cell membrane-coated self-amplified nanosystem as a nanovaccine to boost the therapeutic effect of anti-PD-L1 antibody. Bioact. Mater. 21, 299–312. 10.1016/j.bioactmat.2022.08.028 36157245 PMC9478499

[B80] Limagne E. Thibaudin M. Nuttin L. Spill A. Derangère V. Fumet J.-D. (2019). Trifluridine/tipiracil plus oxaliplatin improves PD-1 blockade in colorectal cancer by inducing immunogenic cell death and depleting macrophages. Cancer Immunol. Res. 7, 1958–1969. 10.1158/2326-6066.CIR-19-0228 31611243

[B81] Liu J. Zhao R. Jiang X. Li Z. Zhang B. (2021). Progress on the application of bortezomib and bortezomib-based nanoformulations. Biomolecules 12, 51. 10.3390/biom12010051 35053199 PMC8773474

[B82] Liu P. Zhao L. Pol J. Levesque S. Petrazzuolo A. Pfirschke C. (2019). Crizotinib-induced immunogenic cell death in non-small cell lung cancer. Nat. Commun. 10, 1486. 10.1038/s41467-019-09415-3 30940805 PMC6445096

[B83] Loi S. Dushyanthen S. Beavis P. A. Salgado R. Denkert C. Savas P. (2016). RAS/MAPK activation is associated with reduced tumor-infiltrating lymphocytes in triple-negative breast cancer: Therapeutic cooperation between MEK and PD-1/PD-L1 immune checkpoint inhibitors. Clin. Cancer Res. 22, 1499–1509. 10.1158/1078-0432.CCR-15-1125.F.D 26515496 PMC4794351

[B84] Luo R. Firat E. Gaedicke S. Guffart E. Watanabe T. Niedermann G. (2019). Cisplatin facilitates radiation-induced abscopal effects in conjunction with PD-1 checkpoint blockade through CXCR3/CXCL10-mediated T-cell recruitment. Clin. Cancer Res. 25, 7243–7255. 10.1158/1078-0432.CCR-19-1344 31506388

[B85] Mahtani R. L. Parisi M. Glück S. Ni Q. Park S. Pelletier C. (2018). Comparative effectiveness of early-line nab-paclitaxel vs. paclitaxel in patients with metastatic breast cancer: A US community-based real-world analysis. Cancer Manag. Res. 10, 249–256. 10.2147/CMAR.S150960 29445301 PMC5808700

[B86] Martins I. Kepp O. Schlemmer F. Adjemian S. Tailler M. Shen S. (2011). Restoration of the immunogenicity of cisplatin-induced cancer cell death by endoplasmic reticulum stress. Oncogene 30, 1147–1158. 10.1038/onc.2010.500 21151176

[B87] Martins I. Tesniere A. Kepp O. Michaud M. Schlemmer F. Senovilla L. (2009). Chemotherapy induces ATP release from tumor cells. Cell. Cycle 8, 3723–3728. 10.4161/cc.8.22.10026 19855167

[B88] Mastria E. M. Cai L. Y. Kan M. J. Li X. Schaal J. L. Fiering S. (2018). Nanoparticle formulation improves doxorubicin efficacy by enhancing host antitumor immunity. J. Control. Release 269, 364–373. 10.1016/j.jconrel.2017.11.021 29146246 PMC6475912

[B89] Mattarollo S. R. Loi S. Duret H. Ma Y. Zitvogel L. Smyth M. J. (2011). Pivotal role of innate and adaptive immunity in anthracycline chemotherapy of established tumors. Cancer Res. 71, 4809–4820. 10.1158/0008-5472.CAN-11-0753 21646474

[B90] Mcquade R. M. Stojanovska V. Bornstein J. C. Nurgali K. (2017). Colorectal cancer chemotherapy: The evolution of treatment and new approaches. Curr. Med. Chem. 24, 1537–1557. 10.2174/0929867324666170111152436 28079003

[B91] Messmer M. N. Snyder A. G. Oberst A. (2019). Comparing the effects of different cell death programs in tumor progression and immunotherapy. Cell. Death Differ. 26, 115–129. 10.1038/s41418-018-0214-4 30341424 PMC6294769

[B92] Michaud M. Martins I. Sukkurwala A. Q. Adjemian S. Ma Y. Pellegatti P. (2011). Autophagy-dependent anticancer immune responses induced by chemotherapeutic agents in mice. Science 334, 1573–1577. 10.1126/science.1208347 22174255

[B93] Modest D. P. Pant S. Sartore-Bianchi A. (2019). Treatment sequencing in metastatic colorectal cancer. Eur. J. Cancer 109, 70–83. 10.1016/j.ejca.2018.12.019 30690295

[B94] Monk B. J. Facciabene A. Brady W. E. Aghajanian C. A. Fracasso P. M. Walker J. L. (2017). Integrative development of a TLR8 agonist for ovarian cancer chemoimmunotherapy. Clin. Cancer Res. 23, 1955–1966. 10.1158/1078-0432.CCR-16-1453 27702821 PMC5437973

[B95] Nagata S. Tanaka M. (2017). Programmed cell death and the immune system. Nat. Rev. Immunol. 17, 333–340. 10.1038/nri.2016.153 28163302

[B96] Nayagom B. Amara I. Habiballah M. Amrouche F. Beaune P. De Waziers I. (2019). Immunogenic cell death in a combined synergic gene- and immune-therapy against cancer. Oncoimmunology 8, e1667743. 10.1080/2162402X.2019.1667743 31741770 PMC6844315

[B97] Nikanjam M. Patel H. Kurzrock R. (2017). Dosing immunotherapy combinations: Analysis of 3,526 patients for toxicity and response patterns. Oncoimmunology 6, e1338997. 10.1080/2162402X.2017.1338997 28920006 PMC5593707

[B98] Ning X. Wang Y. Jing M. Sha M. Lv M. Gao P. (2019). Apoptotic caspases suppress type I interferon production via the cleavage of cGAS, MAVS, and IRF3. Mol. Cell. 74, 19–31.e7. 10.1016/j.molcel.2019.02.013 30878284

[B99] Obeid M. Tesniere A. Ghiringhelli F. Fimia G. M. Apetoh L. Perfettini J.-L. (2007). Calreticulin exposure dictates the immunogenicity of cancer cell death. Nat. Med. 13, 54–61. 10.1038/nm1523 17187072

[B100] Patnaik A. Rosen L. S. Tolaney S. M. Tolcher A. W. Goldman J. W. Gandhi L. (2016). Efficacy and safety of abemaciclib, an inhibitor of CDK4 and CDK6, for patients with breast cancer, non-small cell lung cancer, and other solid tumors. Cancer Discov. 6, 740–753. 10.1158/2159-8290.CD-16-0095 27217383

[B101] Petrazzuolo A. Perez-Lanzon M. Martins I. Liu P. Kepp O. Minard-Colin V. (2021). Pharmacological inhibitors of anaplastic lymphoma kinase (ALK) induce immunogenic cell death through on-target effects. Cell. Death Dis. 12, 713. 10.1038/s41419-021-03997-x 34272360 PMC8285454

[B102] Petroni G. Buqué A. Zitvogel L. Kroemer G. Galluzzi L. (2021). Immunomodulation by targeted anticancer agents. Cancer Cell. 39, 310–345. 10.1016/j.ccell.2020.11.009 33338426

[B103] Petroni G. Formenti S. C. Chen-Kiang S. Galluzzi L. (2020). Immunomodulation by anticancer cell cycle inhibitors. Nat. Rev. Immunol. 20, 669–679. 10.1038/s41577-020-0300-y 32346095 PMC7584736

[B104] Poupon L. Kerckhove N. Vein J. Lamoine S. Authier N. Busserolles J. (2015). Minimizing chemotherapy-induced peripheral neuropathy: Preclinical and clinical development of new perspectives. Expert Opin. Drug Saf. 14, 1269–1282. 10.1517/14740338.2015.1056777 26058312

[B105] Pozzi C. Cuomo A. Spadoni I. Magni E. Silvola A. Conte A. (2016). The EGFR-specific antibody cetuximab combined with chemotherapy triggers immunogenic cell death. Nat. Med. 22, 624–631. 10.1038/nm.4078 27135741

[B106] Qin J. Kunda N. M. Qiao G. Tulla K. Prabhakar B. S. Maker A. V. (2019). Vaccination with mitoxantrone-treated primary colon cancer cells enhances tumor-infiltrating lymphocytes and clinical responses in colorectal liver metastases. J. Surg. Res. 233, 57–64. 10.1016/j.jss.2018.07.068 30502288

[B107] Qu L. Li K. Liu K. Hu W. (2022). Effects of gemcitabine and oxaliplatin combined with apatinib on immune function and levels of SIL-2R and sicAM-1 in patients with gallbladder cancer. Comput. Intell. Neurosci. 2022, 4959840. 10.1155/2022/4959840 36059420 PMC9436547

[B108] Roberts N. B. Alqazzaz A. Hwang J. R. Qi X. Keegan A. D. Kim A. J. (2018). Oxaliplatin disrupts pathological features of glioma cells and associated macrophages independent of apoptosis induction. J. Neuro-oncology 140, 497–507. 10.1007/s11060-018-2979-1 PMC6580860 30132163

[B109] Rongvaux A. Jackson R. Harman C. C. D. Li T. West A. P. De Zoete M. R. (2014). Apoptotic caspases prevent the induction of type I interferons by mitochondrial DNA. Cell. 159, 1563–1577. 10.1016/j.cell.2014.11.037 25525875 PMC4272443

[B110] Roumenina L. T. Daugan M. V. Petitprez F. Sautès-Fridman C. Fridman W. H. (2019). Context-dependent roles of complement in cancer. Nat. Rev. Cancer 19, 698–715. 10.1038/s41568-019-0210-0 31666715

[B111] Russell J. H. Ley T. J. (2002). Lymphocyte-mediated cytotoxicity. Annu. Rev. Immunol. 20, 323–370. 10.1146/annurev.immunol.20.100201.131730 11861606

[B112] Sagiv-Barfi I. Kohrt H. E. Burckhardt L. Czerwinski D. K. Levy R. (2015). Ibrutinib enhances the antitumor immune response induced by intratumoral injection of a TLR9 ligand in mouse lymphoma. Blood 125, 2079–2086. 10.1182/blood-2014-08-593137 25662332 PMC4375105

[B113] Sałat K. (2020). Chemotherapy-induced peripheral neuropathy-part 2: Focus on the prevention of oxaliplatin-induced neurotoxicity. Pharmacol. Rep. P. R. 72, 508–527. 10.1007/s43440-020-00106-1 PMC7329798 32347537

[B114] Salmon H. Remark R. Gnjatic S. Merad M. (2019). Host tissue determinants of tumour immunity. Nat. Rev. Cancer 19, 215–227. 10.1038/s41568-019-0125-9 30867580 PMC7787168

[B115] Sawaki M. Taira N. Uemura Y. Saito T. Baba S. Kobayashi K. (2020). Randomized controlled trial of trastuzumab with or without chemotherapy for HER2-positive early breast cancer in older patients. J. Clin. Oncol. 38, 3743–3752. 10.1200/JCO.20.00184 32936713

[B116] Schaer D. A. Geeganage S. Amaladas N. Lu Z. H. Rasmussen E. R. Sonyi A. (2019). The folate pathway inhibitor pemetrexed pleiotropically enhances effects of cancer immunotherapy. Clin. Cancer Res. 25, 7175–7188. 10.1158/1078-0432.CCR-19-0433 31409612

[B117] Schiavoni G. Sistigu A. Valentini M. Mattei F. Sestili P. Spadaro F. (2011). Cyclophosphamide synergizes with type I interferons through systemic dendritic cell reactivation and induction of immunogenic tumor apoptosis. Cancer Res. 71, 768–778. 10.1158/0008-5472.CAN-10-2788 21156650

[B118] Schneeweiss A. Michel L. L. Möbus V. Tesch H. Klare P. Hahnen E. (2022). Survival analysis of the randomised phase III GeparOcto trial comparing neoadjuvant chemotherapy of intense dose-dense epirubicin, paclitaxel, cyclophosphamide versus weekly paclitaxel, liposomal doxorubicin (plus carboplatin in triple-negative breast cancer) for patients with high-risk early breast cancer. Eur. J. Cancer 160, 100–111. 10.1016/j.ejca.2021.10.011 34801353

[B119] Seicean S. Seicean A. Plana J. C. Budd G. T. Marwick T. H. (2012). Effect of statin therapy on the risk for incident heart failure in patients with breast cancer receiving anthracycline chemotherapy: An observational clinical cohort study. J. Am. Coll. Cardiol. 60, 2384–2390. 10.1016/j.jacc.2012.07.067 23141499

[B120] Shaffer K. L. Sharma A. Snapp E. L. Hegde R. S. (2005). Regulation of protein compartmentalization expands the diversity of protein function. Dev. Cell. 9, 545–554. 10.1016/j.devcel.2005.09.001 16198296

[B121] Smyth M. J. Ngiow S. F. Ribas A. Teng M. W. L. (2016). Combination cancer immunotherapies tailored to the tumour microenvironment. Nat. Rev. Clin. Oncol. 13, 143–158. 10.1038/nrclinonc.2015.209 26598942

[B122] Snyder A. G. Hubbard N. W. Messmer M. N. Kofman S. B. Hagan C. E. Orozco S. L. (2019). Intratumoral activation of the necroptotic pathway components RIPK1 and RIPK3 potentiates antitumor immunity. Sci. Immunol. 4, eaaw2004. 10.1126/sciimmunol.aaw2004 31227597 PMC6831211

[B123] Song L. Hao Y. Wang C. Han Y. Zhu Y. Feng L. (2022). Liposomal oxaliplatin prodrugs loaded with metformin potentiate immunotherapy for colorectal cancer. J. Control. Release 350, 922–932. 10.1016/j.jconrel.2022.09.013 36108810

[B124] Song Q. Yin Y. Shang L. Wu T. Zhang D. Kong M. (2017). Tumor microenvironment responsive nanogel for the combinatorial antitumor effect of chemotherapy and immunotherapy. Nano Lett. 17, 6366–6375. 10.1021/acs.nanolett.7b03186 28858519

[B125] Spisek R. Charalambous A. Mazumder A. Vesole D. H. Jagannath S. Dhodapkar M. V. (2007). Bortezomib enhances dendritic cell (DC)-mediated induction of immunity to human myeloma via exposure of cell surface heat shock protein 90 on dying tumor cells: Therapeutic implications. Blood 109, 4839–4845. 10.1182/blood-2006-10-054221 17299090 PMC1885516

[B126] Sprooten J. Agostinis P. Garg A. D. (2019). Type I interferons and dendritic cells in cancer immunotherapy. Int. Rev. Cell. Mol. Biol. 348, 217–262. 10.1016/bs.ircmb.2019.06.001 31810554

[B127] Sun F. Cui L. Li T. Chen S. Song J. Li D. (2019). Oxaliplatin induces immunogenic cells death and enhances therapeutic efficacy of checkpoint inhibitor in a model of murine lung carcinoma. J. Recept. Signal Transduct. Res. 39, 208–214. 10.1080/10799893.2019.1655050 31441696

[B128] Tan C. R. C. Abdul-Majeed S. Cael B. Barta S. K. (2019). Clinical pharmacokinetics and pharmacodynamics of bortezomib. Clin. Pharmacokinet. 58, 157–168. 10.1007/s40262-018-0679-9 29802543

[B129] Tatsuno K. Yamazaki T. Hanlon D. Han P. Robinson E. Sobolev O. (2019). Extracorporeal photochemotherapy induces bona fide immunogenic cell death. Cell. Death Dis. 10, 578. 10.1038/s41419-019-1819-3 31371700 PMC6675789

[B130] Tesniere A. Panaretakis T. Kepp O. Apetoh L. Ghiringhelli F. Zitvogel L. (2008). Molecular characteristics of immunogenic cancer cell death. Cell. Death Differ. 15, 3–12. 10.1038/sj.cdd.4402269 18007663

[B131] Trajković S. Dobrić S. Jaćević V. Dragojević-Simić V. Milovanović Z. Dordević A. (2007). Tissue-protective effects of fullerenol C60(OH)24 and amifostine in irradiated rats. Colloids Surfaces. B, Biointerfaces 58, 39–43. 10.1016/j.colsurfb.2007.01.005 17317115

[B132] Vacchelli E. Ma Y. Baracco E. E. Sistigu A. Enot D. P. Pietrocola F. (2015). Chemotherapy-induced antitumor immunity requires formyl peptide receptor 1. Sci. (New York, N.Y.) 350, 972–978. 10.1126/science.aad0779 26516201

[B133] Vaes R. D. W. Hendriks L. E. L. Vooijs M. De Ruysscher D. (2021). Biomarkers of radiotherapy-induced immunogenic cell death. Cells 10, 930. 10.3390/cells10040930 33920544 PMC8073519

[B134] Van Der Voort A. Van Ramshorst M. S. Van Werkhoven E. D. Mandjes I. A. Kemper I. Vulink A. J. (2021). Three-Year follow-up of neoadjuvant chemotherapy with or without anthracyclines in the presence of dual ERBB2 blockade in patients with ERBB2-positive breast cancer: A secondary analysis of the TRAIN-2 randomized, phase 3 trial. JAMA Oncol. 7, 978–984. 10.1001/jamaoncol.2021.1371 34014249 PMC8138752

[B135] Vandivier R. W. Ogden C. A. Fadok V. A. Hoffmann P. R. Brown K. K. Botto M. (2002). Role of surfactant proteins A, D, and C1q in the clearance of apoptotic cells *in vivo* and *in vitro*: Calreticulin and CD91 as a common collectin receptor complex. J. Immunol. 169, 3978–3986. 10.4049/jimmunol.169.7.3978 12244199

[B136] Vanmeerbeek I. Sprooten J. De Ruysscher D. Tejpar S. Vandenberghe P. Fucikova J. (2020). Trial watch: Chemotherapy-induced immunogenic cell death in immuno-oncology. Oncoimmunology 9, 1703449. 10.1080/2162402X.2019.1703449 32002302 PMC6959434

[B137] Vanneman M. Dranoff G. (2012). Combining immunotherapy and targeted therapies in cancer treatment. Nat. Rev. Cancer 12, 237–251. 10.1038/nrc3237 22437869 PMC3967236

[B138] Vanpouille-Box C. Alard A. Aryankalayil M. J. Sarfraz Y. Diamond J. M. Schneider R. J. (2017). DNA exonuclease Trex1 regulates radiotherapy-induced tumour immunogenicity. Nat. Commun. 8, 15618. 10.1038/ncomms15618 28598415 PMC5472757

[B139] Vesely M. D. Kershaw M. H. Schreiber R. D. Smyth M. J. (2011). Natural innate and adaptive immunity to cancer. Annu. Rev. Immunol. 29, 235–271. 10.1146/annurev-immunol-031210-101324 21219185

[B140] Wang Q. Wang Y. Ding J. Wang C. Zhou X. Gao W. (2020). A bioorthogonal system reveals antitumour immune function of pyroptosis. Nature 579, 421–426. 10.1038/s41586-020-2079-1 32188939

[B141] Wang R. Xu X. Li D. Zhang W. Shi X. Xu H. (2022). Smart pH-responsive polyhydralazine/bortezomib nanoparticles for remodeling tumor microenvironment and enhancing chemotherapy. Biomaterials 288, 121737. 10.1016/j.biomaterials.2022.121737 36031455

[B142] Wang Y. Gao W. Shi X. Ding J. Liu W. He H. (2017). Chemotherapy drugs induce pyroptosis through caspase-3 cleavage of a gasdermin. Nature 547, 99–103. 10.1038/nature22393 28459430

[B143] Wang Y. Xie W. Humeau J. Chen G. Liu P. Pol J. (2020). Autophagy induction by thiostrepton improves the efficacy of immunogenic chemotherapy. J. Immunother. Cancer 8, e000462. 10.1136/jitc-2019-000462 32221018 PMC7206967

[B144] Wang Z. Chen J. Hu J. Zhang H. Xu F. He W. (2019). cGAS/STING axis mediates a topoisomerase II inhibitor-induced tumor immunogenicity. J. Clin. Invest. 129, 4850–4862. 10.1172/JCI127471 31408442 PMC6819145

[B145] Wculek S. K. Cueto F. J. Mujal A. M. Melero I. Krummel M. F. Sancho D. (2020). Dendritic cells in cancer immunology and immunotherapy. Nat. Rev. Immunol. 20, 7–24. 10.1038/s41577-019-0210-z 31467405

[B146] Wildiers H. Meyskens T. Marréaud S. Lago L. D. Vuylsteke P. Curigliano G. (2022). Long term outcome data from the EORTC 75111-10114 ETF/BCG randomized phase II study: Pertuzumab and trastuzumab with or without metronomic chemotherapy for older patients with HER2-positive metastatic breast cancer, followed by T-DM1 after progression. Breastedinbg. Scotl. 64, 100–111. 10.1016/j.breast.2022.05.004 PMC9157551 35636341

[B147] Wu B. B. Leung K. T. Poon E. N.-Y. (2022). Mitochondrial-targeted therapy for doxorubicin-induced cardiotoxicity. Int. J. Mol. Sci. 23 (3), 1912. 10.3390/ijms23031912 35163838 PMC8837080

[B148] Wu D. Pusuluri A. Vogus D. Krishnan V. Shields C. W. Kim J. (2020). Design principles of drug combinations for chemotherapy. J. Control. Release 323, 36–46. 10.1016/j.jconrel.2020.04.018 32283210

[B149] Wu J. Waxman D. J. (2018). Immunogenic chemotherapy: Dose and schedule dependence and combination with immunotherapy. Cancer Lett. 419, 210–221. 10.1016/j.canlet.2018.01.050 29414305 PMC5818299

[B150] Wu J. Waxman D. J. (2014). Metronomic cyclophosphamide schedule-dependence of innate immune cell recruitment and tumor regression in an implanted glioma model. Cancer Lett. 353, 272–280. 10.1016/j.canlet.2014.07.033 25069038 PMC4162810

[B151] Xu Q. Chen C. Lin A. Xie Y. (2017). Endoplasmic reticulum stress-mediated membrane expression of CRT/ERp57 induces immunogenic apoptosis in drug-resistant endometrial cancer cells. Oncotarget 8, 58754–58764. 10.18632/oncotarget.17678 28938593 PMC5601689

[B152] Yamaguchi K. Minashi K. Sakai D. Nishina T. Omuro Y. Tsuda M. (2022). Phase IIb study of pembrolizumab combined with S-1 + oxaliplatin or S-1 + cisplatin as first-line chemotherapy for gastric cancer. Cancer Sci. 113, 2814–2827. 10.1111/cas.15462 35701865 PMC9357620

[B153] Yamazaki T. Buqué A. Ames T. D. Galluzzi L. (2020). PT-112 induces immunogenic cell death and synergizes with immune checkpoint blockers in mouse tumor models. Oncoimmunology 9, 1721810. 10.1080/2162402X.2020.1721810 32117585 PMC7028345

[B154] Yang C.-P. H. Horwitz S. B. (2017). Taxol^®^: The first microtubule stabilizing agent. Int. J. Mol. Sci. 18 (8), 1733. 10.3390/ijms18081733 28792473 PMC5578123

[B155] Yang Q. Shi G. Chen X. Lin Y. Cheng L. Jiang Q. (2020). Nanomicelle protects the immune activation effects of Paclitaxel and sensitizes tumors to anti-PD-1 Immunotherapy. Theranostics 10, 8382–8399. 10.7150/thno.45391 32724476 PMC7381738

[B156] Yatim N. Cullen S. Albert M. L. (2017). Dying cells actively regulate adaptive immune responses. Nat. Rev. Immunol. 17, 262–275. 10.1038/nri.2017.9 28287107

[B157] Yu Z. Guo J. Hu M. Gao Y. Huang L. (2020). Icaritin exacerbates mitophagy and synergizes with doxorubicin to induce immunogenic cell death in hepatocellular carcinoma. ACS Nano 14, 4816–4828. 10.1021/acsnano.0c00708 32188241

[B158] Zhang M. Hutter G. Kahn S. A. Azad T. D. Gholamin S. Xu C. Y. (2016). Anti-CD47 treatment stimulates phagocytosis of glioblastoma by M1 and M2 polarized macrophages and promotes M1 polarized macrophages *in vivo* . PloS One 11, e0153550. 10.1371/journal.pone.0153550 27092773 PMC4836698

[B159] Zhang S. Li Y. Liu S. Ma P. Guo M. Zhou E. (2022). Ischemia and reperfusion injury combined with cisplatin induces immunogenic cell death in lung cancer cells. Cell. Death Dis. 13, 764. 10.1038/s41419-022-05176-y 36057637 PMC9440929

[B160] Zhang Y. Mei Q. Liu Y. Li X. Brock M. V. Chen M. (2017). The safety, efficacy, and treatment outcomes of a combination of low-dose decitabine treatment in patients with recurrent ovarian cancer. Oncoimmunology 6, e1323619. 10.1080/2162402X.2017.1323619 28932630 PMC5599090

[B161] Zhao X.-Y. Zhao X.-S. Wang Y.-T. Chen Y.-H. Xu L.-P. Zhang X.-H. (2016). Prophylactic use of low-dose interleukin-2 and the clinical outcomes of hematopoietic stem cell transplantation: A randomized study. Oncoimmunology 5, e1250992. 10.1080/2162402X.2016.1250992 28123892 PMC5215224

[B162] Zhao X. Yang K. Zhao R. Ji T. Wang X. Yang X. (2016). Inducing enhanced immunogenic cell death with nanocarrier-based drug delivery systems for pancreatic cancer therapy. Biomaterials 102, 187–197. 10.1016/j.biomaterials.2016.06.032 27343466

[B163] Zhou F. Feng B. Yu H. Wang D. Wang T. Ma Y. (2019). Tumor microenvironment-activatable prodrug vesicles for nanoenabled cancer chemoimmunotherapy combining immunogenic cell death induction and CD47 blockade. Adv. Mater. 31, e1805888. 10.1002/adma.201805888 30762908

[B164] Zhu H. Shan Y. Ge K. Lu J. Kong W. Jia C. (2020). Oxaliplatin induces immunogenic cell death in hepatocellular carcinoma cells and synergizes with immune checkpoint blockade therapy. Cell. Oncol. Dordr. 43, 1203–1214. 10.1007/s13402-020-00552-2 32797385 PMC12990673

[B165] Zsiros E. Lynam S. Attwood K. M. Wang C. Chilakapati S. Gomez E. C. (2021). Efficacy and safety of pembrolizumab in combination with bevacizumab and oral metronomic cyclophosphamide in the treatment of recurrent ovarian cancer: A phase 2 nonrandomized clinical trial. JAMA Oncol. 7, 78–85. 10.1001/jamaoncol.2020.5945 33211063 PMC7677872

